# Nanoparticles and Nanocarriers for Managing Plant Viral Diseases

**DOI:** 10.3390/plants14203118

**Published:** 2025-10-10

**Authors:** Ubilfrido Vasquez-Gutierrez, Gustavo Alberto Frias-Treviño, Luis Alberto Aguirre-Uribe, Sonia Noemí Ramírez-Barrón, Jesús Mendez-Lozano, Agustín Hernández-Juárez, Hernán García-Ruíz

**Affiliations:** 1 Departamento de Parasitología, Universidad Autónoma Agraria Antonio Narro, Calzada Antonio Narro 1923, Buenavista, Saltillo 25315, Mexico; d_ubilfrido.vazquezG@uaaan.edu.mx (U.V.-G.); servesa_gfriast@hotmail.com (G.A.F.-T.); luisaguirre49@hotmail.com (L.A.A.-U.); 2 Departamento de Ciencias Básicas, Universidad Autónoma Agraria Antonio Narro, Calzada Antonio Narro 1923, Buenavista, Saltillo 25315, Mexico; sonia.rmz.barron@gmail.com; 3 Departamento de Biotecnología Agrícola, CIIDIR Unidad Sinaloa, Instituto Politécnico Nacional, Guasave 81101, Mexico; jmendezl@ipn.mx; 4 Department of Plant Pathology and Nebraska Center for Virology, University of Nebraska-Lincoln, Lincoln, NE 68583, USA

**Keywords:** dsRNA, gene silencing, nanomaterials, RNAi, viral diseases, antiviral silencing

## Abstract

The nourishment of the human population depends on a handful of staple crops, such as maize, rice, wheat, soybeans, potatoes, tomatoes, and cassava. However, all crop plants are affected by at least one virus causing diseases that reduce yield, and in some parts of the world, this leads to food insecurity. Conventional management practices need to be improved to incorporate recent scientific and technological developments such as antiviral gene silencing, the use of double-stranded RNA (dsRNA) to activate an antiviral response, and nanobiotechnology. dsRNA with antiviral activity disrupt viral replication, limit infection, and its use represents a promising option for virus management. However, currently, the biggest limitation for viral diseases management is that dsRNA is unstable in the environment. This review is focused on the potential of nanoparticles and nanocarriers to deliver dsRNA, enhance stability, and activate antiviral gene silencing. Effective carriers include metal-based nanoparticles, including silver, zinc oxide, and copper oxide. The stability of dsRNA and the efficiency of gene-silencing activation are enhanced by nanocarriers, including layered double hydroxides, chitosan, and carbon nanotubes, which protect and transport dsRNA to plant cells. The integration of nanocarriers and gene silencing represents a sustainable, precise, and scalable option for the management of viral diseases in crops. It is essential to continue interdisciplinary research to optimize delivery systems and ensure biosafety in large-scale agricultural applications.

## 1. Introduction

Globally, there are priority crops to meet consumer demand, and according to the FAO https://www.fao.org/faostat/en/#data/QCL (accessed on 16 June 2025), these include wheat, rice, maize, sugarcane, soybeans, cassava, and vegetables of the *Solanaceae* family. The database shows figures for cultivated area, global production, and value in millions of U.S. dollars (USD) of the priority crops between 1994 and 2023 (Table S1: Priority crops 1994–2023).

According to data from 2023 from the World Bank Group https://datacatalog.worldbank.org/search/dataset/0037712 (accessed on 12 May 2025), the world population is 8.061 billion people. The World Health Organization (WHO) states that each person should consume 400 g of fruit and vegetables per day and 45 g of cereals. In 2024 there was a total production of 2.853 billion tons of cereals [1], this only supplied 6.34 billion consumers. Meanwhile, 1.976 billion tons of fruits and vegetables were produced, supplying 4.9175 billion of the world’s population. Thus, the current levels of food production is not sufficient to meet the demand of the world’s population. There are multiple constraints that impact food production, including phytosanitary problems, such as diseases caused by pathogens, including viruses [2].

The impact of viral infections on crop production is not new. Since their initial description, plant viruses have been considered a global problem for agricultural production [3]. Recently, for three different isolates of *Tobamovirus fructirugosum*, (tomato brown rugose fruit virus, ToBRFV), yield losses ranged from 40 to 80% [4,5,6]. However, forecasting production losses is not enough; the search for effective management strategies against viruses is increasingly necessary.

To combat viral diseases, several control strategies have been implemented: (a) recessive or dominant genetic resistance [7], (b) transgenic gene silencing [8], (c) spray-induced gene silencing [9,10], (d) phytovirus-inhibiting nanoparticles [11], and (e) double-stranded RNA (dsRNA) nanocarriers [12,13]. This review discusses the use of nanoparticles and dsRNA nanocarriers to control viral diseases in crops through spray-induced gene silencing. Nanoparticles are materials with at least one dimension in the nanoscale (1–100 nanometers). Nanocarriers are a subtype of nanoparticle that are employed to transport and deliver other substances, such as dsRNA, into plant cells. Consequently, nanocarriers are nanoparticles; however, not all nanoparticles are nanocarriers. Virus-induced gene silencing, host-induced gene silencing, and spray-induced gene silencing have shown promise when integrated with nanotechnology to target viral genomes or replication-associated proteins to suppress viral infections.

## 2. Impact of Viral Infections

The economic impact of viral infections on major crops, according to the FAO [1], is being underestimated due to reports of occasional infections in plants and the presence of asymptomatic or mildly symptomatic species [14]. Over the past 25 years, various epidemics caused by plant viruses have been reported, and some of these continue to pose a global problem that threatens crop production [2,7]. Table 1 shows a list of viral diseases that have caused epidemics in priority crops.

## 3. Emerging Tools for Managing Viruses in Plants

**Figure 1:**
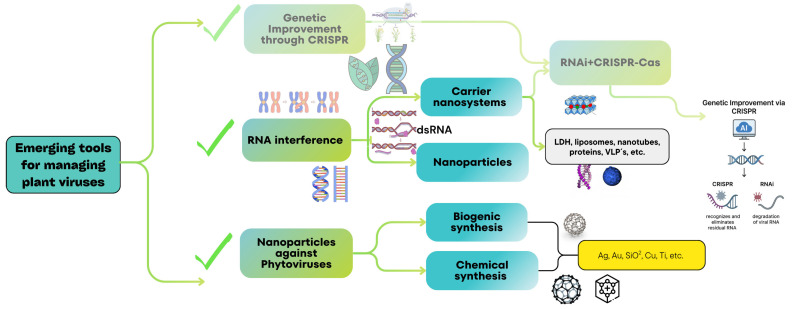
Emerging bionanotechnological tools for managing viral diseases in priority crops.

The management of viral diseases is largely dependent on vector control and on genetic resistance [6]. However, the discovery of gene silencing as a critical component of antiviral defense [42] has led to several biotechnological applications, such as transgenic resistance, and the use of dsRNA to activate antiviral defense. Currently, the biggest limitation in viral diseases management is that dsRNA is unstable in the environment. To eliminate this limitation, nanoparticles and nanocarriers are an option to deliver small interfering RNAs (RNAs) or dsRNA into plant cells [43,44] (Figure 1). Similarly, the gene editing of susceptibility genes to engineer genetic resistance in plants [14] has emerged as a powerful complementation to traditional plant breeding.

## 4. Nanoparticles with Antiviral Activity Against Plant Viruses

Nanoparticles function as protective carriers, preventing the degradation of siRNAs, dsRNA or microRNAs and enhancing their absorption into plant cells to enhance biological activity [45]. Additionally, some nanoparticles such as silver (AgNPs), zinc oxide (ZnONP), and silicon (SiONP) interact with viral proteins, thereby preventing binding, replication, and spread of infection in host plants. These nanoparticles have been effective against common viruses such as TMV and PVY [46,47]. Table 2 summarizes information about nanoparticles that inhibit plant viruses.

The first use of nanoparticles to inhibit plant viruses was carried out with metallic nanoparticles, such as silver, zinc oxide, and silicon, targeting *Tobamovirus tabaci* TMV (tobacco mosaic virus) [46]. These nanoparticles could alter viral activity by interacting with viral particles or enhancing plant immunity. SiO_2_NP deactivated TMV in vitro by causing aggregation and breakage of viral particles while activating the plant defense and growth response instead of increasing plant resistance to infection in vivo. This innovative research established the groundwork for the application of nanotechnology in the treatment of plant viral diseases, thereby demonstrating a substantial advancement in the field of agricultural science [46,47].

Different types of synthesis have been developed to obtain nanoparticles. Chemical synthesis is widely used due to its precision in controlling size and composition. Metallic nanoparticles such as silver and copper (Cu NPs) are synthesized using chemical reduction techniques [48]. However, these methods often involve inorganic reagents, raising concerns about environmental impacts and plant safety [49]. Alternatively, green synthesis has emerged as an environmentally friendly option, using plant extracts or microbial agents to produce nanoparticles; this method reduces toxicity while maintaining efficacy, as biomolecules in natural extracts frequently contribute to antiviral activity [50]. These techniques enable the production of NPs with unique surface properties that enhance interactions with viral particles, thereby improving their antiviral efficacy [51]. However, they may require a significant amount of energy, which limits their scalability. Another promising approach is biogenic synthesis, which harnesses biological entities such as bacteria, fungi, and algae to produce nanoparticles [52]. The latter approach is not only consistent with sustainable practices, but also guarantees plant compatibility, as biosynthesized nanoparticles frequently demonstrate biocompatibility and antiviral activity [13]. The synthesis method selected for the purpose of specific virus control in plants is contingent upon factors such as cost, scalability, and the desired properties of the nanoparticles. The cost of nanoparticle synthesis application in plants is lower than the average cost of antiviral products, which ranges from USD 100 to 500 per liter [53]. To synthesize one liter of Ag NPs at 1000 ppm, USD 0.5 is needed, considering reagents and precursors.

**Table 2:** Nanoparticles used against plant viruses.

Nanoparticle	Viral Species	Crop Host	Context	Reference
Silver (Ag)	*P.phaseoluteum,* BYMV (bean yellow mosaic virus (BYMV)	Broad bean (*Vicia faba* L.)	A total of 100 mg to 200 mg L^−1^ of AgNPs inactivated BYMV when applied 48 and 24 h before and after inoculation. NPs are bioreactive with viral particles, specifically toward the capsid protein, regulate the PR-1 gene related to pathogenesis, and induce the production of enzymes as a defense mechanism in plants.	[54]
Zinc oxide (ZnO)	*B. capsicumhuastecoense,* PHYVV (pepper huasteco yellow vein virus)	Pepper (*Capsicum annum* L.)	NPsZnO at 100 mM and 150 mM reduced disease severity and viral levels, inducing resistance mechanisms in plants through the activation of POD, SOD, and CAT.	[55]
Copper oxide (CuO) through biosynthesis with *Haloxylon salicornicum*	*Alfamovirus AMV,* AMV (alfalfa mosaic virus)	Tobacco (*Nicotiana tabacum* L.)	Foliar applications of CuO NPs (48 h before and after) improved tobacco plant growth, decreased viral symptoms, reduced AMV accumulation levels by 97%, increased the expression of antioxidant enzymes, and increased the expression of genes as an antiviral mechanism. CuO NPs showed high binding energy with viral replication protein 1a.	[56]
Carbon (C_60_), zinc (Zn) and iron (Fe)	*Cucurbit chlorotic yellows virus* (CCYV)	*N. benthamiana*	NPs of C_60_ at 100 mg L^−1^ delayed viral infection for up to 5 days after inoculation. Fe and Zn did not suppress viral progression, while C_60_ regulated the production of defense-related phytohormones (SA and JA).	[57]

SOD: superoxide dismutase; POD: peroxidase; CAT: catalase; SA: salicylic acid; JA: jasmonic acid.

## 5. Mechanisms of Gene Silencing Against Plant Viruses

The reduction or permanent inactivation of gene expression without altering the DNA is considered gene silencing [58]. Gene silencing has natural roles in plant development and protection of the genome [42], is a critical component of antiviral defense [59] and also acts against non-viral pathogens [60].

Gene silencing can occur at the transcriptional (TGS) or post-transcriptional (PTGS) levels [42,61]. Transcriptional gene silencing prevents the synthesis of new mRNA transcripts by methylating the DNA. Post-transcriptional gene silencing refers to the translational repression, cleavage or degradation of mRNAs in cells [42]. Gene silencing is a critical component of antiviral defense, providing resistance against viral infections [61]. Transcriptional gene silencing affects geminiviruses and is triggered by the production of virus-derived small interfering RNAs that mediate the RNA-directed DNA methylation of the viral genome and suppress viral transcription and limit virus replication [62]. RNA viruses and viroids are targeted by post-transcriptional gene silencing [42].

Gene silencing triggered by double-stranded (dsRNA) in the form of stem-loop structures form by virus replication intermediates, bidirectional transcription, or by endogenous RNA-dependent RNA polymerases [63]. DsRNA that is processed into small interfering RNAs that measure 21 to 24 nt (siRNA) by Dicer-like proteins (DCL). Depending on their origin and processing pathway, there are several classes of siRNA in plants, such as micro-RNAs, trans-acting siRNAs, virus-derived siRNAs and others [64]. siRNAs are unwound and loaded into argonaute proteins to form the RNA-induced silencing complex (RISC) [65]. AGO proteins are guided by the siRNA to cleave RNAs with complementary sequences to the siRNA [66]. Silencing is amplified by siRNA-mediated dsRNA synthesis. In some cases, AGO-proteins loaded with siRNAs that are 22 nt long or originate from particular precursors, RNA-dependent RNA polymerases are recruited to synthesize dsRNA from the initial RNA target. The new dsRNAs is processed into secondary siRNAs that downregulate their target in trans, as in trans-acting siRNAs [67]. Proteins SGS2, SGS3 and HEN1 are essential for the production and activity of siRNA involved in gene silencing.

Interestingly, siRNAs derived from the virus downregulate both viral and host RNAs with sequence complementarity [45]. By modifying a viral genome to carry part of a plant gene, gene silencing induced by the virus targets the plant gene of interest through sequence complementarity [68]. This phenomenon is called virus-induced gene silencing (VIGS), and has been used extensively to downregulate plant genes for functional genomic studies and crop improvement [69,70], and to generate resistance against viruses in plants [8]. Recent advances have integrated VIGS with CRISPR/Cas9 for precise gene editing, enhancing its utility for crop improvement and epigenetic studies [68]. However, challenges such as limited viral host range and off-target effects remain areas for optimization [71]. Currently, more than 35 viruses have been used as VIGS vectors. *Potexvirus ecspotati*, PVX (potato virus X), TMV, *Begomovirus solanumaureimusivi*, TGMV (tomato golden mosaic virus) and *Tobravirus tabaci*, TRV (tobacco rattle virus) belong to the first generation of VIGS vectors causing short-term silencing [72,73]. The second generation of VIGS-selected viruses responsible for mild symptoms improved the range of hosts, and their systemic transmission includes meristem infections and fewer viruses in infected plants [74]. Novel advances continue to finetune the use of viruses as vectors to induce silencing and prevent or reduce infection by other viruses [64,75].

Plant siRNAs capable of targeting viral RNA of ToBRFV have been described, suggesting that *S. lycopersicum* encodes mature miRNAs with a protective function [76] and also highlighting the intriguing possibility of using plant endogenous siRNA against viruses.

### 5.1. Endogenous and Virus-Derived Gene Silencing

Endogenous gene silencing involves endogenous RNAs and siRNAs that target cellular endogenous genes. Antiviral gene silencing involves the activation of the pathway by RNA from invading viruses and the generation of siRNAs derived from viral RNA and that target viral RNA [77]. Viruses generate dsRNA as replication intermediates, overlapping translation, or by forming hairpins on single-stranded RNA, all of which may trigger the RNA silencing pathways in the following three-step process: (i) recognition and processing of dsRNA by DICER-like enzymes (DCL) that generate siRNAs measuring 21 to 24 nt; (ii) amplification of the silencing signal by RNA-dependent RNA polymerases, leading to the formation of secondary siRNAs and (iii) the systemic spread of siRNAs through the entire plant [78].

### 5.2. Host-Induced and Spray-Induced Gene Silencing

Host-induced gene silencing (HIGS) is a type of gene silencing that uses siNRNAs in the plant for viral, non-viral pathogens, or insects. HIGS is a transgenic method that provides a source of dsRNA in the form of inverted repeats of hairpins that are processed into siRNAs [79] that target specific genes in the pathogen [80], occasionally moving from plant cells to pathogen cells to downregulate the critical genes involved in virulence or development [81]. HIGS has been established as a powerful and efficient technology for controlling viral diseases in plants [82]. The development of efficient, resistant, and polycistronic miRNA and the fusion of multiple genes into the hairpin RNA has proven to be efficiently successful [78,83]. However, the use of transgene technology to manage viral diseases in commercial agriculture has been limited by negative public perception and regulation [84,85].

An alternative to transgenic technology is spray-induced gene silencing (SIGS) and is emerging as a strategy to activate gene silencing against viral, non-viral diseases and insect pests [86]. SIGS relies on topical application of externally synthesized dsRNA or siRNAs that trigger silencing [79]. This non-transgenic approach has gained attention for its potential in sustainable and ecological crop protection, as it avoids genetic modifications [87]. The topical application of dsRNA has been demonstrated to confer resistance to several viral species, including *T. capsici* PMMoV (pepper mottle mosaic virus) and *Alfamovirus AMV* (AMV) [9,88]. Similarly, infiltration with a syringe or mechanical inoculation with sterile soft brushes facilitate the introduction of dsRNA into plants. However, these methods are difficult to scale up in the field and greenhouse, which represents a limitation [87,88]. These difficulties led to the incorporation of nanostructures to coat dsRNA for direct applications in greenhouses and to streamline gene silencing in plants [9,10,89].

## 6. Nanocarriers for dsRNA Delivery

Insects, fungi, oomycetes, bacteria and nematodes present serious phytosanitary threats to crops. Recent studies with double-layered hydroxide nanosheets (LDH) have demonstrated a prolonged resistance against specific viruses when dsRNA is applied to crops [90]. This technique, known as “BioClay,” holds great promise for broad-spectrum crop protection by enabling precise, environmentally sustainable antiviral treatments that significantly reduce the need for active chemical ingredients [9,10,68,91]. Despite its potential, there is still a limited body of research on the use of nanostructures for dsRNA delivery aimed at achieving long-lasting virus resistance in plants [92].

Meanwhile, the use of dsRNA-carrying nanosystems for the silencing of pests, including fungi, nematodes, and bacteria, has achieved a novel and significant scope [90]. These systems have the ability to transport different biomolecules such as DNA, miRNA, siRNA, and ribonucleoproteins, which has led to them being considered as non-cytotoxic and biocompatible vectors [93].

The nanocarriers with dsRNA, commonly referred to as RNA-based pesticides, function inhibiting gene expression in pest, thereby impairing their development or leading to their death [94]. They are currently regarded as the third revolution in pesticides technology due to their high specificity and effectiveness in controlling a wide range of pathogens and insects pest [71]. However, disadvantages have emerged that affect their efficiency, durability in the host, instability in the dsRNA environment, and low accumulation. The low stability and efficiency of RNA delivery have restricted the development of dsRNA pesticides. Recent studies have focused on impregnating dsRNA on to nanomaterials to improve stability [9,95,96]. This has led to the development of dsRNA delivery systems to plant cells and the streamlining of gene silencing [92,97]. The obstacle for these nanosystems, as well as for nanoparticles, is to achieve stability, allowing them to be introduced into cell walls and viral proteins [98], where siRNA could exert an effective mechanism of action [99]. Therefore, this action will depend on the nanometric size of the carriers, shapes, surface charges, and functionality for pest attacks [93]. According to this, properties could be adjusted to achieve the effective release of the biomolecule at the intracellular level [99].

### 6.1. Functionalization of Nanocarriers Against Insects Pests

The use of chitosan nanocarriers protect the dsRNA from pH degradation and hydrolysis, regulate the target genes and cause a high mortality for *Helicoverpa armigera*; compared to other pests, the designed dsRNAs were specific and showed no off-target effects [100]. It has been mentioned that nanocarriers spray-induced and nanocarrier-delivered gene silencing with dsRNA on *Sogatella furcifera* produce a high mortality (>60%) and a reduced ecdysis, although the attacked genes and methods for delivery could vary with respect to the species [101]. Similarly, nanotransporters have been delivered on *Adelphocoris suturalis,* targeting JH signal genes, where they effectively inhibited oviposition, and allowed the development of a new RNA fertility inhibitor to control *A. suturalis* populations [102]. Alternatively, hollow rough-surfaced mesoporous silica was potentiated with dsRNA and an Imidacloprid insecticide, which increased the stability and toxicity of imidacloprid [103]. Chitosan impregnated with dsRNA against *Spodoptera frugiperda* and *Ostrinia nubilalis* improves the efficiency of insect target RNA interference [104,105], and chitosan derivatives combined with sodium ripolyphosphate show efficacy against *Aedes aegypti* [105,106]. Spraying with a star polycation nanocarrier (SPc) impregnated with dsRNA increased the ability of dsRNA to penetrate the body wall of fleas, and dsRNA silenced the expression of target genes [107]. In addition, Sun et al. [108] used a mixture of dsRNA with SPc to target the cytochrome P450 monooxygenase gene CYP15C1, which increases the mortality of *Chilo supperssalis* larvae. Gold-coated nanopolymers can carry luciferases and induce RNA silencing in *S. frugiperda* transcription [109]. Ref. [110] administered *Cre* recombinases in *Zea mays* cells coated in gold nanomaterials that harbored loxP sites from a selection gene and reporter gene.

Nanocarriers (NCs) loaded with dsRNA have demonstrated significant potential in mitigating diseases and pest insects in economically important crops such as tomatoes, eggplants, and peppers [44]. Per se, they provide stability and efficient delivery of dsRNA to plant cells, overcoming the challenges of environmental degradation and improving the persistence of gene-silencing effects [92].

These biotechnological advances in nanocarriers continue to progress steadily, from chemical–metallic synthesis to biogenic synthesis [111], but advances in in vitro dsRNA synthesis have not lagged behind [112]. The obstacle to scaling up dsRNA production for agricultural applications is a challenge due to high investment costs and low yields [82]. In vitro transcription is fast but expensive, while chemistry is more adaptable for producing short siRNAs [113]. The use of microorganisms to synthesize dsRNA has shown greater relevance due to its cost-effectiveness and optimization for production in biological factories [111]. Bacteria and yeasts of the genera *Escherichia* sp. [113], *Pseudomonas* sp. [114], *Bacillus* sp. [115], *Lactobacillus* sp. [116], and *Corynebacterium* sp. [117] synthesize dsRNA and use it for gene silencing in plant diseases.

### 6.2. Functionalization of Nanocarriers Against Pathogens

The functionalization of nanocarriers with ligands or peptides allows for greater specificity and improves the prolonged release of dsRNA against fungal pathogens [118]. The development of nanofungicides of functionalized carbon dots with dsRNA against *Phytophthora* sp. has improved the effect of spray-induced gene silencing [119]. The selection of chitosan, polyethyleneimine, protamine, carbon quantum dot, polyamidoamine, and chitosan/SPc complex as dsRNA carriers achieved long-lasting protection of up to 20 days against rice sheath blight caused by *Rhizoctonia solani* in rice plants [120]. Chitosan polyplex/dsRNA nanoparticles targeting the RiABCG6.3 gene were also used for *Rhizophoraceae irregularis*. It is possible that RiABCG6.3 forms the appressorium in *R. irregularis*, and dsRNA silences the RiABCG6.3 transcripts [121]. The use of small layered double hydroxide (sLDH) loaded with dsRNAs targeting endogenous genes (BcBmp1, BcBmp3 and BcPls1), decreased the symptoms of the disease compared to controls. The sLDH-dsRNA complexes showed a better protection of the plants at 27 days after inoculation [122]. In a recent study, self-assembled nanoparticles formed by ε-poly-L-lysine (ε-PL) and carboxymethylchitosan were synthesized. The *R. solani* glycosyl hydrolase family 1 (RsGH1), which functions as a cell-wall-degrading enzyme, was selected as a possible RNAi target gene for the management of *R. solani* AG3 TB. The application of ε-poly-L-lysine (ε-PL) and carboxymethylchitosan improved the RNAi efficiency of ds RsGH1 and prolonged its protective duration in maize and rice crops. Ds RNA for RsGH1 derived from *R. solani* AG3 TB also exhibited broad-spectrum activity against *R. solani* AG1-IA in rice and maize plants. In this study, a self-assembled multicomponent nanofungicide based on dsRNA and nanotransporters was designed [123].

The effective scaling of these nanosystems administered to roots and plants offers a sustainable alternative for crop disease management and the replacement of chemical molecules [118].

Although the development of RNA-carrying nanosystems to silence genes in nematodes, viruses and bacteria is not well defined, studies have been conducted on the silencing of the pat-10 and unc-87 genes of *Pratylenchus thornei* and *P. zeae*, causing paralysis and uncoordinated movements in both species, although with a higher incidence in *P. thornei* [124]. Other studies reported lethal genes (Bxy1177, Bxy1239, Bxy1104, Bxy667, and BxyAK1) and tested them by feeding the pine wood nematode (PWN), *Bursaphelenchus xylophilus*, with a dsRNA-modified endophytic fungus (*F. babinda*). Nematodes that consumed fungi expressing dsL1177 and dsAK1 showed a substantial decline over time [125]. These findings provide new insights and a practical basis for employing dsRNA expressed by endophytic fungi in sustainable pest control strategies.

sRNAi impregnated in fluorescent polyethyleneimine (PE)-functionalized gold nanoparticles enhanced gene silencing through the administration of RNA in leaf cells targeting the ArWRKY1 gene, resulting in improved resistance to *P syringae* [126].

Application by spraying, infiltration, root soaking, and internalization of pollen with chitosan quaternary ammonium chloride (HACC) reduced the replication rate of TMV; dsRNA-HACC facilitated transport, resulting in the silencing of homologous molecules [127]. The efficiency of *P. yitiburosum* (potato virus Y, PVY) gene silencing has been investigated by topical application of chitosan quaternary ammonium salt (CQAS) nanotransporters on *N. benthamiana* plant roots [128]. Nanoliposomes used as carrier molecules for the biological antiviral molecule 7 quercetin with Hsp70 genes improved the inhibitory effect by 34 and 42% at the gene and protein levels, respectively. This study suggested that the use of nanomaterials reduced the dose of active ingredients and improved efficacy for effective disease management [129]. These studies show the efficacy of nanocarriers in the delivery of dsRNA into cells, and RNAi, when induced by dsRNA, improves gene silencing against plant viruses.

DsRNA nanocarriers to induce gene silencing against viruses in plants include carbon nanotubes, nanocapsules, mesoporous nanoparticles, proteins, liposomes, polymers, metals, silicas, double-layered hydroxides and carbide whiskers (Table 3).

These systems enable the precise targeting and effective silencing of viral genes, positioning them as a foundational component of integrated strategies for the management of viral infections in plants [127,128,130,131,132].

**Table 3:** Nanocarriers of dsRNA for silencing viral infections, and against non-viral pathogens or pests.

Nanomaterial	Feature	Pathogen/Pest	Crops	Ref.
Chitosan	Cellulose structure; negative charges confer greater connection to dsRNA.	*Spodoptera frugiperda*	*Cicer arietinum*	[104,106,118]
Poly-l-arginine (PLR-polyplex), Au nanoparticles functionalized with poly-L-arginine (PLR/Au NPs)	Exposure of PLR-dsRNA in a stable *S. frugiperda* Sf9 cell line (Sf9_LUC) for 72 h inhibited the luciferase gene by 58%.	*S. frugiperda*	In vitro	[109]
Chitosan nanoparticles (CNPs)	dsRNAs against *Helicoverpa armigera* JHAMT and ACHE target genes loaded on cationic CNPS effectively protected from nuclease degradation and insect intestinal pH. CNP-ache-dsRNA at a low dose (0.028 g/ha) in chickpea showed a reduction in damage to the pods with high yields.	*Helicoverpa armigera*	*Cicer arietinum*	[100]
Chitosan polyplexes	dsRNAs impregnated in chitosan NPs regulate the key HCC gene and activate the CDE pathway, a component that improves the efficiency of RNAi.	*Tetranychus cinnabarinus* Boisduval	In vitro	[132]
Chitosan nanohydrogel	Knockdown potential of formulated dsRNA targeting ECR gene was evaluated through bioassay. A higher mortality rate of ≥80% was achieved through a low concentration of formulated dsRNA.	*Bemisia tabaci*	In vitro	[133]
Cationic nucleocap (nanodetergent)	Action on the chitinase gene of the midgut and cuticle target gene.	*Aphis glycines*	*Glycine max*	[134]
Star polycation nanocarrier (SPc)	Cationic dendrimer, which condenses random amino acids absorbed by endocytosis.	*Chilo supperssalis*	*Glycine max*	[108]
Not loaded onto nanocarriers. Topical application	The application of dsRNA targeting the EcR and USP did not promote the silencing of genes involved in growth and development. On the contrary, degradation of dsRNA was found in aphid salivary secretions, as well as in hemolymph from the hemocoel of the body. There was no expression of genes related to RNA dicer-2 argonaute-2, r2d2, and sid-1.	*Acyrthosiphon pisum*	In vitro	[135]
Chitosan-based polymer	Nanocomposites were tested by injection and orally to improve the stability of dsRNA targeting a gene encoding the third-instar larval protein (OnLgl). The combination of chitosan polymers and dsRNA improved the ECB silencing.	*Ostrinia nubilalis*	Ex vivo	[105]
Cellfectin II (CFII) transfection reagent	The formulation of dsRNA with CFII protected it from degradation by endonucleases. Exposure of dsRNA-CFII in *S. frugiperda* cells produced a decrease in endogenous genes (iap) and also had a negative effect on growth and mortality.	*S. frugiperda*	In vitro	[106]
Alginate-chitosan	The incubation of conidia in dsRNA deformed the germ tube of *M. oryzae*. Foliar spraying with alginate–chitosan NCs impregnated with dsRNA suppressed disease progression in the cereal.	*Magnaporthe oryzae*	*Brachypodium distachyon*	[136]
Cationic poly-aspartic acid-derived polymer (CPP6)	dsRNA was administered to *Pratylenchus thornei* and *P. zeae* stages by ingestion. The silencing targeted pat-10 and unc-87 genes, which caused paralysis and uncoordinated movements in both species, although to a greater extent in *P. thornei*. A greater reduction in gene transcription and reproduction was observed, indicating that *P. thornei* may be more susceptible to RNAi.	*Xanthomonas oryzae pv. oryzae* (Xoo)	*Arabidopsis* sp. and *Oryza sativa*	[137]
Gold nanoparticles functioned with fluorescent polyethyleneimine (PEI) (PEI-AuNPs)	siRNAs with PEI-AuNPs improved loading and delivery; fluorescence allowed siRNA traceability in cells. The silenced plants showed a higher resistance to *P. syringae*, showing a lower amount of bacteria and ROS.	*Pseudomonas syringae*	*Arabidopsis thaliana* (Col-0)	[126]
Was not used	dsRNA was administered to *Pratylenchus thornei* and *P. zeae* stages by ingestion. The silencing targeted pat-10 and unc-87 genes, which caused paralysis and uncoordinated movements in both species, although to a greater extent in *P. thornei*. A greater reduction in gene transcription and reproduction was observed, indicating greater susceptibility to RNAi.	*Pratylenchus thornei* and *P. zeae*	Tissue disks, carrot (*Daucus carota*)	[124]
Spherical protein nanoparticles (SNPs)	SNPs with diameters of 100–200 nm, formed by thermal annealing of TMGMV coat proteins, were impregnated with dsRNA. Topical application of dsRNA-SNP to a transgenic line of *C. elegans* triggers RNAi after ingestion and persists for 180 h.	*Caenorhabditis elegans*	In vitro	[138]
Was not used	dsRNA targeting lethal genes (Bxy1177, Bxy1239, Bxy1104, Bxy667 and BxyAK1) were synthesized. *B. xylophilus* nematodes were immersed on a dsRNA solution and fed with dsRNA-modified *F. babinda*. The genes were deleted using both methods. Nematodes that consumed fungi expressing dsL1177 and dsAK1 showed substantial decreases over time.	*Bursaphelenchus xylophilus*	In vitro	[125]
Double laminar hydroxide (LDH)	Nano sheets with clay-like anionic cations with layered structures similar to brucite, which facilitate dsRNA adhesion through an ion exchange mechanism.	*P. phaseovulgaris*, BCMV (bean common mosaic virus)	*Vigna unguiculata*	[9,10]
		*P. phaseovulgaris* BCMV (bean common mosaic virus)	*Nicotiana benthamiana*	[91]
KH)_9_ péptide Bp100	dsRNA delivery system based on the ionic complex of dsRNA and a peptide. The results showed that the complex was absorbed by leaf cells and induced rapid and efficient regulation of exogenous and endogenous genes.	N/E	*A. thaliana*	[130]
Single-walled carbon nanotubes	For use as an RNAi delivery system in plants. The system protects RNAi from degradation, resulting in mRNA elimination within one day with 95% efficiency.	Development of a platform for the administration of dsRNA using nanotubes	mGFP5 *N. benthamiana*	[12]
Quaternary ammonium salt of chitosan (HACC)	HACC, complexed with selected siRNA, targeted genes of the CP of the TMV and the TMV RdRP1 to form siRNA-HACC.	*T. tabaci*, TMV (tobacco mosaic virus)	*N. benthamiana*	[127]
Chitosan quaternary ammonium salt (CQAS), amine functionalized silica nano powder (ASNP), and carbon quantum dots (CQD)	The application of ASNP in root immersion effectively silenced genes in plants and provided 14 days of protection against PVY.	*P. yitiburosum* (PVY).	*Solanum tubersoum*	[128]
Lipid-modified polyethyleneimine (lmPEI)	The application of the lmPEI NCs for dsRNA (250bp) silenced RNA polymerase and CP genes of GLRaV-3. The structure protected the dsRNA from degradation by ribonucleases. A single foliar application of lmPEI reduced the viral titer, and multiple applications maintained the basal viral load in the vine and berries.	*Ampelovirus trivitis*, GLRaV-3 (grapevine leafroll-associated virus 3)	*Vitis vinifera* L.	[139]
Quercetin nanoliposomes	The field application of nanoliposomes (Nbhsp70er-1 and Nbhsp70c-A) released quercetin and inhibited the expression of the hsp70 gene by 42%. The efficiency of the TMV control under field conditions was 38%.	*T. tabaci,* TMV (tobacco mosaic virus)	*N. benthamiana*	[129]
Double-laminar hydroxide (LDH)	RNAds were loaded into monodisperse, biodegradable hexagonal LDH layers, which provided high stability. Topical spraying was performed on cells, leaves, petiole adsorption, and tomato root immersion. It provided protection against crown and root rot for 60 days.	*F. oxysporum* f. sp. *radicis-lycopersici* (FORL)	*Solanum lycopersicum* L.	[140]
Functionalized carbon points (CDs)	The dual treatment with dsRNA-CDs showed a 90% protective effect in plants. The elution of CDs enhances the internalization of dsR-NA into recipient cells. First application of a nano-administration system to improve the effect of the SIGS.	*Phytophthora infestans*, *P. sojae* and *P. capsici*	*N. benthamiana*	[119]
Chitosan (CS), polyethyleneimine (PEI), protamine, carbon quantum dot (CQD), polyamidoamine (PAMAM)	Spraying of CS, PEI, CQD, PAMAM and CSC with dsRNA protected rice pods for up to 20 days against R. solani. The NCs improved the carrying capacity and stability of dsRNA.	*Rhizoctonia solani*	Rice sheaths (*Oryza sativa* L.)	[120]
Small-layered double hydroxide (sLDH)	The spraying of in vitro synthesized dsRNA molecules (BcBmp1, BcBmp3 and BcPls1) loaded in LDHs, decreased the symptoms of the disease, and showed greater protection in inoculated plants at 27 days later.	*Botrytis cinerea*	Lettuce (*Lactuca sativa*)	[122]
Chitosan polyplex/dsRNA nanoparticles	Chitosan can transport dsRNA, and successfully silence the *RiABCG6.3* gene in *R. irregularis* compared to naked RNAds.	*Rhizophagus irregularis*	*Astragalus sinicus*	[121]
Nanoparticles formed by ε-poly-L-lysine (ε-PL) and carboxymethylchitosan (CMCS)	RsGH1 was identified as a potential siRNA target for controlling *R. solani*. Self-assembled nanoparticles ε-PL and CMCS can load dsRNA efficiently. ε-PL@CMCS effectively protects RNase A from dsRNA degradation and markedly improves RNAi efficiency. ds RsGH1 exhibited broad-spectrum activity against *R. solani* AG1-IA in rice and maize plants.	*R. solani*	*Nicotiana tabacum* (*N. tabacum*)	[123]

RDR1: RNA-dependent RNA polymerase 1 (RdRP1); HCC: clathrin heavy chain; JHAMP: juvenile hormone methyltransferase; ACHE: acetylcholinesterase; CDE: clathrin-pendant endocytosis; EcR: ecdysone receptor; USP: ultraspiracle; TMGMV: tobacco mild green mosaic virus; TMV: tobacco mosaic virus; RsGH1: glycosyl hydrolase family 1; CP: coat protein; endogenous plant genes flowering locus T (FT); phytochrome interaction factor 4 (PIF4); NCs: nanocarriers; SIGS: spray-induced gene silencing.

## 7. Entry and Mode of Action of dsRNA Nanocarriers

**Figure 2:**
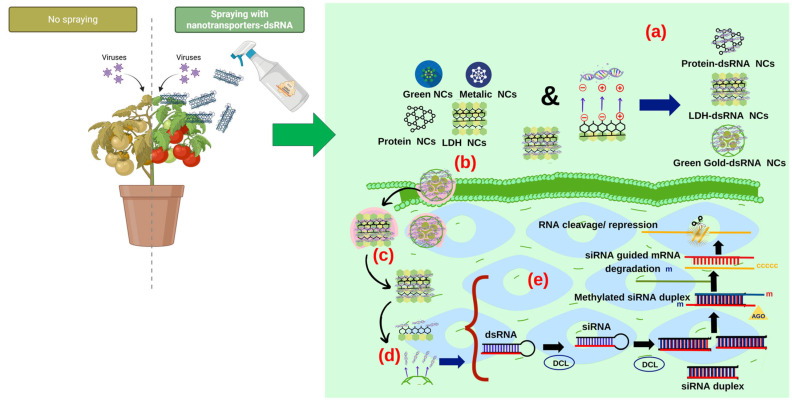
Release of dsRNA from nanocarriers and activation of gene silencing. Spraying is carried out using green synthesis nanomaterials, metals, proteins, and double-layered hydroxide. (**a**) The binding of dsRNA to nanocomposites is achieved through covalent and/or covalent bonds, which allows for the binding, delivery, and release of NCs with dsRNA. Double-stranded RNA is synthesized through a repeated inverted sequence of hairpin RNA by RNA polymerase and is then impregnated into double-layered hydroxide NCs and green gold nanoparticles. (**b**) These nanoparticles are internalized by plant cells via endocytosis, where the positively charged dsRNAs interacts with the membrane, which produces the lysis of the endosome. (**c**) Endomic escape happens to ensure that the dsRNA reaches the cytoplasm and exerts gene silencing. (**d**) The release of dsRNA occurs in the cell, activates gene silencing, and results in the formation of interfering siRNAs against viruses. (**e**) DCL cleaves the dsRNA to produce a siRNA duplex. This duplex is methylated by the HEN protein. AGO proteins bind one strand of the siRNA duplex and downregulate the RNA target. Green NCs: green synthesis nanocarriers; Metallic NCs: metallic nanocarriers; protein nanocarriers; double-layered hydroxide (LDH) nanocarriers; dsRNA: double-stranded RNA; DCL: dicer enzyme; siRNA: small interfering RNA; mRNA: messenger RNA. This figure was created using BioRender and Microsoft PowerPoint 2025. “https://BioRender.com (accessed on 8 July 2025)”.

Assembly with nucleic acids through electrostatic interactions allows for the protection of dsRNA from nuclease degradation, enhances dsRNA uptake via endocytosis activation and improves RNAi efficiency for gene silencing. Nanoparticles have been applied for conferring resistance against plant viral diseases [101,141]. This has provided effective scaling for dsRNA delivery in the host and streamlined gene silencing against plant viruses [142]. The process and mechanism of dsRNA delivery mediated by nanocarriers can be summarized in four steps (Figure 2): (a) binding or encapsulation of dsRNA, (b) cellular uptake, (c) endosomal escape and (d) release of nucleic acids or degradation of nanocomplexes.

(a) Binding or encapsulation of dsRNA: double-stranded RNA (dsRNA) is loaded into nanoparticles using techniques such as electrostatic interactions (non-covalent bonds), covalent bonds, or physical encapsulation to improve stability and protect against enzymatic degradation [73]. Covalent interactions occur when two non-metallic atoms share electrons. These bonds are strong and stable and form the basis of organic molecules. This occurs with biogenic and green synthetic nanoparticles [143]. Non-covalent interactions, on the other hand, are attractive forces between atoms that do not share electrons but are attracted by opposite charges. They are weaker than covalent interactions, but the effect of several non-covalent interactions achieves the stabilization of a dsRNA molecule [144]. Some types of non-covalent interactions involved in dsRNA binding with nanomaterials are ion–ion interactions (ion groups with opposite charges), dipole–dipole interactions (alignment of molecules with positive and negative poles), ion–dipole interactions (an ion with a polar molecule), Van der Waals forces (electrostatic density in molecules), and hydrogen bonds (interaction of H+ bound to an electronegative heteroatom) [145]. Nanoparticles, which are composed of lipids, polymers, or inorganic materials, serve as carriers to improve the bioavailability of dsRNA [146]. In most cases, positively charged NCs can self-assemble with negatively charged dsRNA through electrostatic interaction, forming dsRNA/nanocarrier complexes with hydrogen bonds and Van der Waals forces, which contribute to the self-assembly process of complexes [141]. An example of this could be chitosan, which has a large number of positively charged amino groups under acidic conditions that interact electrostatically with negatively charged dsRNA, forming dsRNA/chitosan complexes [147].
(b) Cellular uptake: dsRNA-loaded nanoparticles are internalized into target cells via endocytosis, with surface modifications such as the selection of ligands that recognize specific cell receptors [148]. This improves cellular uptake, ensuring delivery to the desired intracellular locations [149]. Positively charged dsRNA complexes facilitate interaction with the membrane, allowing entry through receptor-mediated endocytosis [150]. After cellular uptake, dsRNA complexes are coated by vesicles called endosomes in the membranes. In the case of cationic polymers, this leads to endosome lysis [151].
(c) Endosomal escape: once internalized, nanoparticles escape from endosomal compartments to avoid degradation; this is achieved through the proton sponge effect, membrane fusion, or endosomal disruption [145]. Efficient endosomal escape is crucial to ensure that dsRNA reaches the cytoplasm for its biological activity, gene silencing [152].
(d) Release of nucleic acids or degradation of nano-complexes: after escaping, dsRNA is released through cellular environmental stimuli, e.g., pH, redox conditions, and degradation of the nanoparticle matrix (phytochemical compounds) [153]. Controlled release allows dsRNA to interact with RNA interference pathways or other cellular targets for antiviral action [154]. Subsequently, the dsRNA/nanocarrier complex delivered by late endosomes is dispersed, after which the dsRNA is released from the nanocarrier to activate RNAi and exert biological effects on viral infections [92]. The mechanism of dsRNA release has not yet been confirmed. One mechanism of dsRNA release from the nanotransporter is the slow displacement process, while the second mechanism is based on the response to intracellular stimuli such as acidic pH and cytosolic reductants [92]. For example, poly(β-aminoester) nanocarriers respond to changes in environmental pH, whereas those containing disulfide (SS) are stimulated by intracellular glutathione redox reactions, and these changes aid in the release of dsRNA/siRNA [155,156]. Release of dsRNA in the cell activates gene silencing and results in the formation of small interfering siRNAs [83]. The mechanism of gene silencing occurs as explained in Section 5.1 (Figure 2e).

## 8. Prospects for Nanoparticles and dsRNA Nanocarriers Against Viruses

The current research focuses on improving the delivery of dsRNA to plant cells. However, studies should not only focus on RNA impregnation, but also on the type of nanomaterial to be used. Several metallic nanoparticles such as gold and silver have been evaluated to improve sRNA adhesion, but the waste produced by nanomaterial synthesis is very important because of environmental pollution [54]. Therefore, future research should not only focus on the synthesis of nanomaterials using green plant extracts, but also extrapolate the use of biogenic compounds such as fungi, bacteria, and even nematodes [52]. The use of biological organisms to synthesize silver, copper, zinc, and gold would have a lesser impact on living beings. However, incorporating microorganisms that are pathogenic to humans and plants would have an impact on the added value for controlling plant viruses [26]. Naimi-Shamel et al. [157] explored the use of *F. oxysporum* for the synthesis of gold nanoparticles. Reference [158] used *F. pseudonygamai* TB-13c to synthesize silver nanoparticles and test their antibacterial efficiency against *Klebsiella pneumoniae*, *Pseudomonas aeruginosa*, and *Staphylococcus aureus*. Despite this, the green synthesis of silver nanoparticles using walnut shell extracts could serve as an effective reducing agent for obtaining NCs with dsRNA [159].

The synthesis of nanomaterials through “green” methods holds significant ecological value [143]. This approach utilizes plant extracts, microorganisms, or other agents of biological origin that are used for synthesis to produce nanoparticles, which minimizes their ecological footprint compared to conventional chemical synthesis processes [50]. When impregnated with dsRNA, these nanoparticles are effective while reducing toxicity to non-target organisms and the surrounding environment [49,52]. Ecologically synthesized zinc oxide nanoparticles have been shown to have antiviral activity while promoting plant immunity [46]. These methods align with the principles of sustainable agriculture, ensuring that biotechnological interventions do not compromise environmental health [87]. However, to improve the efficacy and scalability of dsRNA nanomaterials for virus control in plants, the following strategies need to be explored: (i) hybrid nanomaterials: develop hybrid systems that combine different nanomaterials to improve stability, targeted delivery, and release of double-stranded RNA in plant cells. (ii) Precision engineering: incorporating artificial intelligence and machine learning to design more effective dsRNA sequences and NCs tailored to specific interactions between plants and viruses. (iii) Field-scale implementation: advances in spray formulations and encapsulation techniques for large-scale agricultural applications (greenhouse and open field). (iv) Integrative approaches: combining dsRNA nanomaterials with other biocontrol methods, such as beneficial microorganisms, to create synergistic effects against viral infections. (v) Regulatory and economic considerations: addressing regulatory frameworks and reducing production costs to make dsRNA nanomaterials accessible for widespread agricultural use. These innovations could revolutionize plant disease management, leading to improved crop yields, reduced economic losses, and increased food security, while maintaining ecological balance.

## 9. Future Directions for the Management of Plant Viruses

**Figure 3:**
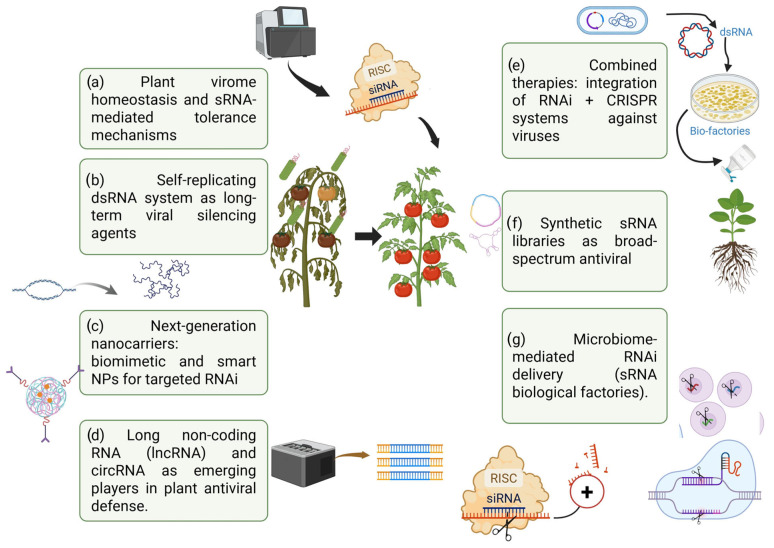
Future directions for modulating plant virus–host interactions toward sustainable homeostasis. RISC: RNA-induced silencing complex; sRNA: small RNA; NPs: nanoparticles; RNAi: RNA interference; circRNA: circular RNA. IncRNA: RNA molecules longer than 200 nucleotides that do not encode proteins and play a role in regulating gene expression. This figure was created using BioRender. “https://BioRender.com (accessed on 8 July 2025)”.

Due to climate change and the excessive use of chemical compounds, there are increasing cases of plant viruses with accelerated replication, mutations, and new pathogenic variants that affect priority crops. It is worth considering that, perhaps, the solution is not the management of control through chemical and biological strategies, but to innovate tactics where viruses and the host can interact in constant homeostasis. Therefore, seven directions are proposed for possible future research that could modulate viral diseases in crops (Figure 3).

(a) **Plant virome homeostasis and viral tolerance mechanisms through RNA.** Management should not only focus on resistance (virus elimination) but also explore natural “tolerance” mechanisms where mRNA pathways stop viral infections without causing severe damage, maintaining “virome homeostasis.” RNAi can shift from being a resistance strategy to a tool that induces tolerance against mixed viral infections.
(b) **Self-replicating dsRNA systems as long-term viral silencing agents.** The administration of SIGS and dsRNA in plants is important, but the use of self-replicating RNA replicons (such as the minimal replicons of alphavirus) to produce dsRNA within the plant after a single treatment remains to be explored. This could prolong protection without the use of genetically modified organisms and reduce the frequency of application. Some preliminary studies suggest this idea, but very few relate it directly to virus control in plants.
(c) **Next-generation nanocarriers: biomimetic and intelligent nanoparticles for targeted RNAi.** The idea of using biomimetic nanoparticles that mimic viruses or target ligands to recognize infected cells, release dsRNA specifically in infected tissues, and reduce off-target effects. The emergence of smart reactive delivery systems (activated by pH, ROS, and viral protease activity) could have antiviral uses in plants, although these have not yet been described.
(d) **RNA long non-coding RNAs (lncRNAs) and circRNAs as emerging players in plant antiviral defense.** In recent years, long non-coding RNAs (lncRNAs) and circular RNAs (circRNAs) have emerged as new layers of regulation in the antiviral response of plants. Although attention has traditionally focused on small RNAs (sRNAs), recent re-search suggests that lncRNAs and circRNAs play critical roles in modulating gene expression, acting as miRNA sponges, epigenetic regulators, or mRNA stability modulators, thereby altering the outcomes of viral infection [160,161]. Stability, especially in the case of circRNAs, confers an additional advantage against viral degradation mechanisms. These findings open up new possibilities for the design of bio-technological viral resistance strategies based on the silencing, tolerance, or modulation of non-coding RNA regulatory networks [142]. Looking ahead, targeted manipulation of lncRNA and circRNA could offer complementary approaches to traditional RNAi methods, providing more durable and specific alternatives for protecting crops against viral infections [162]. This emerging area of research promises to transform the current understanding of the plant virome and the engineering of antiviral resistance.
(e) **Combination therapies: integration of RNAi + CRISPR systems against viruses.** The combination of RNAi-based strategies and CRISPR-Cas13 systems represents a promising avenue for strengthening antiviral resistance in plants, using combination therapies that integrate RNA interference with CRISPR-Cas-based gene editing technologies [163]. RNAi has proven to be an effective tool for the targeted degradation of viral RNA using siRNA and miRNA, while the CRISPR-Cas13 system offers the ability to recognize and cut viral RNA sequences in a specific manner. The combination of both platforms would allow for a double antiviral blockade: RNAi would decrease initial viral accumulation, and CRISPR-Cas13 would effectively eliminate residual viral RNAs, reducing the likelihood of mutational escape. In addition, the use of multiplexed systems would allow multiple viruses or variants to be attacked simultaneously, a critical aspect in the face of mixed infections or complex viromes [164]. Although still in the experimental stages, this combined approach promises to revolutionize antiviral resistance strategies in agricultural crops, providing more robust, durable, and specific solutions [89].
(f) **Engineering synthetic sRNA libraries (artificial sRNA groups) for broad-spectrum viral protection.** Is dsRNA necessary for a virus? We should aim to develop “cocktail libraries” of artificial siRNA or miRNA targeting multiple virus families. This would allow us to predict virus mutations (quasispecies) and design groups of small RNAs that “cover” the future evolution of the virus (preventing viral escape).
(g) **RNAi administration mediated by biological siRNA factories.** Use endophytic bacteria or fungi to produce and distribute dsRNA within the plant. Emerging studies explore genetically modified symbionts to express antiviral RNA [113]. Bacteria have been engineered for effective RNAi-mediated control of mosquito larvae [165]. This highlights the innovative use of genetically modified microorganisms as biofactories for sRNA production and delivery.

Together, these perspectives not only open new routes for research in plant virus management, but also consolidate the foundations for the development of innovative and sustainable solutions, thus setting a promising course for the upcoming scientific challenges.

## 10. Conclusions

Tools based on the use of nanosystems and gene silencing via siRNAs are effective and sustainable bio-nanotechnological alternatives. The incorporation of nanotechnology to deliver and stabilize dsRNA molecules improves the efficiency, durability, and specificity of antiviral control without requiring permanent genetic modifications in plants. The recognition of regulatory players such as lncRNAs and circRNAs opens up unexplored horizons for understanding and modulating the plant antiviral response from a more comprehensive perspective. Looking ahead, the combination of RNAi systems with CRISPR-Cas13 technologies, high-throughput sequencing to monitor viral susceptibility genes, and the integration of AI promises more robust, specific, and adaptable antiviral therapies, even against complex viromes or emerging mutants. Scientific advances are bringing us closer to developing dsRNA synthesized in biofactories for gene silencing of multiple viral species. The integration of these technologies into bioformulation platforms, applicable via spraying or bioremediation, represents a new era for antiviral management without resorting to transgenic crops. This review highlights the urgent need to continue developing and adopting innovative, multidisciplinary approaches that will advance toward a more resilient, safe, and biologically integrated agriculture in the face of post-pandemic viral threats.

## Figures and Tables

**Table 1:** Plant diseases and the viruses that cause them in priority crops.

Disease	Host	Symptoms	Common Name and Viral Species	Distribution	Transmission Type	Annual Impact	Reference
Cassava mosaic disease ^a^	Cassava (*Manihot esculenta*)	Yellowing, mosaics, mottling, distortion of leaves and tubers	*Begomovirus manihotis,* ACMV (African cassava mosaic virus); *B. manihotisafricaense*, EACMV/MW (East African cassava mosaic virus); *B. manihotiscameroonense,* EACMCMV (East African cassava mosaic Cameroon virus. *B. manihotisindianense,* ICMV (Indian cassava mosaic virus); *B. manihotiskenyaense,* EACMKV (East African cassava mosaic Kenya virus); *B. manihotismadagascarense* CMMGV (cassava mosaic Madagascar virus); *B. manihotismalawiense,* EACMMV (East African cassava mosaic Malawi virus); *B. manihotiszanzibarense,* EACMZV (East African cassava mosaic Zanzibar vírus); *B. stanleyi,* SLCMV/LK (Sri Lankan cassava mosaic virus); *B. warburgi,* SACMV (South African cassava mosaic virus)	African continent and Indian subcontinent	*Bemisia tabaci* and infected cuttings	USD 1.9–2.7 billion	[15,16]
Cassava brown streak disease ^a^	Cassava (*Manihot esculenta*)	Chlorosis in leaves, brown streaks on stems, and dry, hard rot in roots	*Ipomovirus brunusmanihotis,* CBSV (cassava brown streak vírus); *I. manihotis,* UCBSV (Ugandan cassava brown streak virus)	East, Central, and Southern Africa	*Bemisia tabaci* and possibly by aphids.	Losses are estimated at USD 750 million annually	[17,18]
Dwarf corn mosaic ^a^	Corn (*Zea mays* L.)	Mosaic and streaks on leaves and reduction in size	*Potyvirus zeananus,* MDMV (maize dwarf mosaic vírus)	All over the world	Aphid species: *Rhopalosiphum maidis* (corn leaf aphid), *Myzus persicae* (green peach aphid), *Brevicoryne* brassicae (cabbage aphid), *Aphis fabae* (black bean aphid), *Acyrthosiphon pisum*, *Aphis gossypii* (Cotton aphid).	Up to 70% loss in production, producing USD 30 billion	[19]
Maize lethal necrosis diseases ^a^	Corn (*Zea mays* L.)	Stunted growth, chlorotic streaks and spots on leaves, and necrosis (tissue death)	*Machlomovirus zeae, MCMV* (maize chlorotic mottle virus); *P. sacchari,* SCMV (sugarcane mosaic vírus; *Tritimovirus tritici, WSMV* (wheat streak mosaic virus);	Southeast Asia and South America, Eastern Sub-Saharan Africa	Aphid species: MCMV; mite (*Aceria tosichella*); SCMV: *Hysteroneura setariae;* WSMV: *Aceria tosichella Keifer*	USD 52 million in Kenya alone. Losses in yield due to co-infection of these viral species of up to 90%.	[20]
Potato necrosis and discoloration syndrome ^1, a^	Potato (*Solanum tuberosum* L.)	Spotting, mosaics, necrosis in leaf veins, reduced growth, and defoliation	*P. duobatatae,* SPV 2 (sweet potato virus 2)*; P. yituberosi,* PVY (potato virus and serotypes C, N, NTN, O, strain C and Chinese isolate)	Potato-producing regions around the world	Aphid: *Myzus persicae.*	Estimated yield losses of 40 to 70%	[21,22]
Rice blast disease ^a^	Arroz (*Oryza sativa* L.)	Stunted growth, yellow discoloration of leaves, reduced branching, and poor grain development	*Waikavirus oryzae* RTSV (rice tungro spherical virus); *Tungrovirus oryzae*) RTBV (rice tungro bacilliform virus)	Southwest and East Asia	Cicadellidae: *Nephotettix virescens*	USD 1 billion in annual losses	[23]
Yellow mottle disease of rice ^1, d^	Rice (*Oryza sativa* L.)	Yellowing, spotting, stunted growth, reduced tillering, and spikelet sterility	*Sobemovirus RYMV* (rice yellow mottle virus)	East, West, and Southern African countries	Beetles (Chrysomelidae) mechanically, through contact between infected plant roots	Up to 70% loss in yield	[24]
Wheat streak mosaic disease ^1, a, d^	Wheat (*T. aestivum* L., *H. vulgare* L.)	Mosaic patterns, growth retardation.	(*Tritimovirus tritici,* WSMV (wheat streak mosaic virus); *Poacevirus tritici,* TriMV (Triticum mosaic virus); *Emaravirus tritici*, HPWMoV (high plains wheat mosaic virus);	Eastern Europe, the Middle East, Mexico, Argentina, Australia, and Canada	Wheat mite (*Aceria tosichella*), seeds, and wind	Yield losses that can reach up to 100%	[25,26]
Wheat yellow dwarf disease ^1, d^	Wheat (*Triticum aestivum*)	Yellowing, reddening and growth retardation	*Luteovirus* sp., BYD (barley yellow dwarf virus-kerII, kerIII, MAV, PAS, PAV, SGV, RPV, RMV, GPV). *Polerovirus* sp. strains: RPS and RPV	Europe, Middle East, Asia, Asia, Oceania, North, Central and South America, North Africa, Sub-Saharan Africa	Aphids, especially *Rhopalosiphum padi* (PAV, CYDV), *R. maidis* (RMV), *Sitobion avenae* (MAV) and *Schizaphis graminium* (SGV).	Yield losses of up to 84%, with annual costs of approximately USD 220 billion	[27]
Cucumber mosaic disease ^b^	Tomato (*Solanum lycopersicum* L.) Chile (*Capsicum* spp.)	Mosaics, stunted growth, leaf curling, distorted or reduced fruit.	(*Cucumovirus CMV* (cucumber mosaic virus)	Tropical and subtropical areas with favorable conditions for growing these species	By aphids. Around 75 species and by seeds of plant species	Reduces production yield by 10 to 40%	[28]
Tomato spotted wilt disease ^1, a^	Tomato (*Solanum lycopersicum* L.) Chile (*Capsicum* spp.)	Tanning, necrosis (tissue death), rings, or concentric spots	*Orthotospovirus tomatomaculae,* TSWV (tomato spotted wilt virus)	Tropical and subtropical areas of the world that are conducive to cultivation	Thrips (*Frankiniella occidentalis*).	Tomato crops have suffered losses ranging from 50 to 90%, amounting to an economic cost of USD 1 billion per year.	[29]
Tomato yellow leaf curl disease ^1, a^	Tomato (*Solanum lycopersicum* L.) Chile (*Capsicum* spp.)	Delayed growth, yellowing and curling of leaves, reduced fruit production	*B. coheni,* TYLCV (tomato yellow leaf curl virus)	Asia, the Middle East, North and South America, North Africa, and Sub-Saharan Africa	*Bemisia tabaci*	Yield losses of 11 to 33%, representing USD 247,000 per hectare	[30]
Pepino mosaic disease ^1, a^	Tomato (*Solanum lycopersicum* L.) Chile (*Capsicum* spp.)	Stunted growth, distorted or curled leaves with yellow or brown spots and mosaics	*Potexvirus pepini,* PepMV (*Pepino mosaic vírus).*	North and South America, Europe, the Middle East, and South Africa	Mechanical, through contact between infected and healthy plants, grafts, slight transmission by seeds	Up to 38% loss in crop production	[31]
Tomato rugose virus ^1, b, c^	Tomato (*Solanum lycopersicum* L.), (*Capsicum* spp.) and Solanum melongena.	Mosaics, yellowing, blistering on leaves, and brown roughness on fruits	*Tobamovirus fructirugosum,* ToBRFV (tomato brown rugose fruit virus)	Worldwide distribution in arable areas	Mechanical, due to contact between plants, machinery, and work equipment. Seeds and occasional reports of *Tuta absoluta*. Contaminated soil and substrate	Yield losses of 40 to 90%	[5,6,32]
Tomato fruit spot disease^, d^	Tomato (*Solanum lycopersicum* L.)	Irregular ripening, spots on fruit, hollows, and dark spots	*Blunervirus solani,* TFBV (tomato fruit blotch virus)	Italy, Europe (Greece, Portugal, Slovenia, Spain, Switzerland) South America (Brazil) and Oceania (Australia)	Red mite (*Aculops lycopersici*)	Not estimated	[33,34,35]
Mosaic disease ^a, 1, b^	Tomato (*Solanum lycopersicum* L.) y (*Capsicum* spp.)	Mosaic patterns, mottling, and discoloration on leaves and fruits	*T. viridimaculae,* CGMMV (cucumber green mottle mosaic virus)	Worldwide distribution in arable areas	Seeds, contaminated soil, and mechanics through contact between plants and tools	Production losses of 5 to 40%	[36]
Bean pod mottling disease ^1, c, d^	Soybean (*Glycine max* L.)	Spots on bean pods, distortion, green and light spots, reduction in pod size and number	*Comovirus siliquae,* BPMV (bean pod mottle virus)	North America: Southern and Southeastern United States	Beetle (*Cerotoma trifurcata*), low transmission by seed and infectious sap	Losses of 3 to 52% in global production	[37]
Tobacco ring spot disease ^a, c, d^	Soybean (*Glycine max* L.)	Chlorotic and necrotic rings, which often resemble an oak leaf	*Nepovirus nicotianae,* TRSV (tobacco ringspot virus)	Global distribution. Strong presence in North America, the US Midwest, Ontario, Canada, Australia, China, and Russia	Nematodes: *Xiphinema americanum;* mechanical transmission; infected seeds	30 to 48% loss in yield	[38]
Viral yellowing disease 30 to 48% yield losses ^a, c, d^	Sugar beet (*Beta vulgaris*)	Yellowing, discoloration, thickening, brittleness, and possible necrosis	*Closterovirus flavibetae,* BYV (beet yellows virus)	Europe, North and South America, Asia, and Australia	Aphid: *Myzus persicae*	Yield losses of 28 to 50% in production	[39,40]
Sugarcane mosaic disease ^a, c, d^	Sugarcane (*Saccharum officinarum*)	Yellowing of central veins, dark green mosaic patterns on leaves. Leaf drying and stunted growth	*P. sacchari,* SMV (sugarcane mosaic virus); *Poacevirus sacchari,* MSSMV (sugarcane streak mosaic virus); *Polerovirus SCYLV*, (sugarcane yellow leaf virus)	Asia, Africa, the Middle East, and Central America	Aphid: *Melanaphis sacchari*, *Dactynotus ambrosiae, Hysteroneura setariae, Longiunguis sacchari, Rhopalosiphum maidis* and *Toxoptera graminum*	Yield losses of 10 to 50%, in some cases up to 80%	[41]

^1^ global and recurring; ^a^ Epidemic virus; ^b^ pandemic virus, ^c^ virus with global priority and emerging threat; ^d^ endemic. bd: billions of dollars.

## Data Availability

The underlying data of this manuscript are available upon reasonable request from the authors.

## Supplementary Materials

The following supporting information can be downloaded at https://www.mdpi.com/article/10.3390/plants14203118/s1, Table S1: Priority crops 1994–2023.

## References

[1] Organización Organización de las Naciones Unidas para la Alimentación y la Agricultura (FAO) FAOSTAT: Crops and Livestock Products. https://www.fao.org/faostat/en/#data/QCL.

[2] Jones R.A.C. (2021). Global Plant Virus Disease Pandemics and Epidemics. Plants.

[3] Rao G.P., Reddy M.G. (2020). Overview of yield losses due to plant viruses. Applied Plant Virology.

[4] Vasquez-Gutierrez U., Frías-Treviño G.A., Delgado-Ortiz J.C., Aguirre-Uribe L.A., Flores-Olivas A. (2023). Severity of Tomato brown rugose fruit virus in tomato (*Solanum lycopersicum* L.) from a region of Coahuila, México. Int. J. Hortic. Agric. Food Sci..

[5] Vásquez-Gutiérrez U., Frías-Treviño G.A., López-López H., Delgado-Ortiz J.C., Aguirre-Uribe L.A., Flores-Olivas A. (2024). Evaluation of the Pathogenicity of Three Isolates of Tomato brown rugose fruit virus in tomato plants (*Solanum lycopersicum* L.) from Coahuila, Mexico. Rev. Bio Cienc..

[6] Gutiérrez U.V., Treviño G.A.F., Ortiz J.C.D., Uribe L.A.A., Olivas A.F., Beache M.B., Castillo F.D.H. (2024). Chlorine Dioxide: Antiviral That Reduces the Spread of ToBRFV in Tomato (*Solanum lycopersicum* L.) Plants. Viruses.

[7] Vásquez G.U., Delgado-Ortiz J.C., Frías-Treviño G.A., Aguirre-Uribe L.A., Flores-Olivas A. (2025). Tobamovirus fructirugosum an emerging disease: Review and current situation in Mexico. Mex. J. Phytopathol..

[8] Akbar S., Wei Y., Zhang M.-Q. (2022). RNA Interference: Promising Approach to Combat Plant Viruses. Int. J. Mol. Sci..

[9] Mitter N., Worrall E.A., Robinson K.E., Li P., Jain R.G., Taochy C., Fletcher S.J., Carroll B.J., Lu G.Q., Xu Z.P. (2017). Clay nanosheets for topical delivery of RNAi for sustained protection against plant viruses. Nat. Plants.

[10] Mitter N., Worrall E.A., Robinson K.E., Xu Z.P., Carroll B.J. (2017). Induction of virus resistance by exogenous application of double-stranded RNA. Curr. Opin. Virol..

[11] Eid N.A., Ibrahim A.M.H., Elsharawy A.A., Salem K.F.M., Kumar P., Dubey R.C. (2025). Nanofertilizers for plant viral disease management. Nanofertilizers for Sustainable Agriculture.

[12] Demirer G.S., Zhang H., Goh N.S., Pinals R.L., Chang R., Landry M.P. (2020). Carbon nanocarriers deliver siRNA to intact plant cells for efficient gene knockdown. Sci. Adv..

[13] Rani S., Kumar P., Dahiya P., Dang A.S., Suneja P. (2022). Biogenic synthesis of zinc nanoparticles, their applications, and toxicity prospects. Front. Microbiol..

[14] Garcia-Ruiz H., Szurek B., Van den Ackerveken G. (2021). Stop Helping Pathogens: Engineering Plant Susceptibility Genes for Durable Resistance. Curr. Opin. Biotechnol..

[15] Alabi O.J., Kumar P.L., Naidu R.A. (2011). Cassava Mosaic Disease: A Curse to Food Security in Sub-Saharan Africa. APSnet Features.

[16] Eni A.O., Efekemo O.P., Onile-ere O.A., Pita J.S. (2021). South West and North Central Nigeria: Assessment of Cassava Mosaic Disease and Field Status of African Cassava Mosaic Virus and East African Cassava Mosaic Virus. Ann. Appl. Biol..

[17] Patil B.L., Legg J.P., Kanju E., Fauquet C.M. (2015). Cassava Brown Streak Disease: A Threat to Food Security in Africa. J. Gen. Virol..

[18] Munguti F.M., Nyaboga E.N., Kilalo D.C., Yegon H.K., Macharia I., Mwango’mbe A.W. (2023). Survey of cassava brown streak disease and association of factors influencing its epidemics in smallholder cassava cropping systems of coastal Kenya. Front. Sustain. Food Syst..

[19] Kannan M., Ismail I., Bunawan H. (2018). Maize Dwarf Mosaic Virus: From Genome to Disease Management. Viruses.

[20] Mahuku G., Lockhart B.E., Wanjala B., Jones M.W., Kimunye J.N., Stewart L.R., Cassone B.L., Sevgan S., Nyasani J.O., Kusia E. (2015). Maize lethal necrosis (MLN), an emerging threat to maize-based food security in sub-Saharan Africa. Phytopathology.

[21] Gray S., De Boer S., Lorenzen J., Karasev A., Whitworth J., Nolte P., Singh R., Boucher A., Xu H. (2010). Potato virus Y: An evolving concern for potato crops in the United States and Canada. Plant Dis..

[22] Karasev A.V., Gray S.M. (2013). Continuous and emerging challenges of potato virus Y in potato. Annu. Rev. Phytopathol..

[23] Nihad S.A.I., Manidas A.C., Hasan K., Hasan M.A.I., Honey O., Latif M.A. (2021). Genetic variability, heritability, genetic advance and phylogenetic relationship between rice tungro virus resistant and susceptible genotypes revealed by morphological traits and SSR markers. Curr. Plant Biol..

[24] Pinel-Galzi A., Traoré O., Séré Y., Hébrard E., Fargette D. (2015). The biogeography of viral emergence: Rice yellow mottle virus as a case study. Curr. Opin. Virol..

[25] Singhal P., Nabi S.U., Yadav M.K., Dubey A. (2021). Mixed infection of plant viruses: Diagnostics, interactions and impact on host. J. Plant Dis. Prot..

[26] Tatineni S., Hein G.L. (2023). Plant viruses of agricultural importance: Current and future perspectives of virus disease management strategies. Phytopathology.

[27] Nancarrow N., Aftab M., Hollaway G., Rodoni B., Trębicki P. (2021). Yield Losses Caused by Barley Yellow Dwarf Virus-PAV Infection in Wheat and Barley: A Three-Year Field Study in South-Eastern Australia. Microorganisms.

[28] Ashwathappa K.V., Krishna Reddy M., Venkataravanappa V., Madhavi Reddy K., Hemachandra Reddy P., Lakshminarayana Reddy C.N. (2021). Genome characterization and host range studies of Cucumber mosaic virus belonging to the Subgroup IB infecting chilli in India and screening of chilli genotypes for identification of resistance. Virus Dis..

[29] Sevik M.A., Arli-Sokmen M. (2012). Estimation of the effect of Tomato spotted wilt virus (TSWV) infection on some yield components of tomato. Phytoparasitica.

[30] Walls J., Rajotte E., Rosa C. (2019). The past, present, and future of barley yellow dwarf management. Agriculture.

[31] Hanssen I.M., Thomma B.P.H.J. (2010). Pepino mosaic virus: A successful pathogen that rapidly evolved from emerging to endemic in tomato crops. Mol. Plant Pathol..

[32] Caruso A.G., Tortorici S., Davino S., Bertacca S., Ragona A., Lo Verde G., Panno S. (2024). The invasive tomato pest Tuta absoluta can transmit the emergent tomato brown rugose fruit virus. Entomol. Gen..

[33] Ciuffo M., Kinoti W.M., Tiberini A., Forgia M., Tomassoli L., Constable F.E., Turina M. (2020). A new blunervirus infects tomato crops in Italy and Australia. Arch. Virol..

[34] Nakasu E.Y., Nagata T., Inoue-Nagata A.K. (2022). First report of tomato fruit blotch virus infecting tomatoes in Brazil. Plant Dis..

[35] Beris D., Galeou A., Kektsidou O., Varveri C. (2023). First report of Tomato fruit blotch fruit virus infecting tomato in Greece. New Dis. Rep..

[36] Dombrovsky A., Tran-Nguyen L.T.T., Jones R.A.C. (2017). Cucumber green mottle mosaic virus: Rapidly Increasing Global Distribution, Etiology, Epidemiology, and Management. Annu. Rev. Phytopathol..

[37] Giesler L.J., Ghabrial S.A., Hunt T.E., Hill J.H. (2002). Bean pod mottle virus: A threat to US soybean production. Plant Dis..

[38] Hill J.H., Whitham S.A. (2014). Control of virus diseases in soybeans. Advances in Virus Research.

[39] Werker A.R., Dewar A., Harrington R. (1998). Modelling the incidence of virus yellows in sugar beet in the UK in relation to numbers of migrating *Myzus persicae*. J. Appl. Ecol..

[40] Hossain R., Menzel W., Lachmann C., Varrelmann M. (2021). New insights into virus yellows distribution in Europe and effects of beet yellows virus, beet mild yellowing virus, and beet chlorosis virus on sugar beet yield following field inoculation. Plant Pathol..

[41] Putra L.K., Kristini A., Jati W.W. (2023). Viral diseases of sugarcane in Indonesia: Occurrence notes, pathogenic characteristics and management strategies. Proceedings of the 5th International Conference on Agriculture and Life Science 2021 (ICALS 2021).

[42] Lopez-Gomollon S., Baulcombe D.C. (2022). Roles of RNA Silencing in Viral and Non-Viral Plant Immunity and in the Crosstalk between Disease Resistance Systems. Nat. Rev. Mol. Cell Biol..

[43] Mujtaba M., Wang D., Carvalho L.B., Oliveira J.L., Espirito Santo Pereira A.D., Sharif R., Fraceto L.F. (2021). Nanocarrier-Mediated Delivery of miRNA, RNAi, and CRISPR-Cas for Plant Protection: Current Trends and Future Directions. ACS Agric. Sci. Technol..

[44] Thanjavur N., Ankireddy S.R., Rayi R. (2024). Nanotechnology Advancements in Detecting Pathogenic Human RNA Viruses. Recent Developments in Nanomaterial-Based Sensing of Human Pathogens.

[45] Zhao Y., Zhou Y., Xu J., Fan S., Zhu N., Meng Q., Dai S., Yuan X. (2024). Cross-Kingdom RNA Transport Based on Extracellular Vesicles Provides Innovative Tools for Plant Protection. Plants.

[46] Cai L., Liu C., Fan G., Liu C., Sun X. (2019). Preventing Viral Disease by ZnONPs through Directly Deactivating TMV and Activating Plant Immunity in *Nicotiana benthamiana*. Environ. Sci. Nano.

[47] Dutta P., Kumari A., Mahanta M., Biswas K.K., Dudkiewicz A., Thakuria D., Abdelrhim A.S., Singh S.B., Muthukrishnan G., Sabarinathan K.G. (2022). Advances in Nanotechnology as a Potential Alternative for Plant Viral Disease Management. Front. Microbiol..

[48] Kumari S., Raturi S., Kulshrestha S., Chauhan K., Dhingra S., András K., Singh T. (2023). A Comprehensive Review on Various Techniques Used for Synthesizing Nanoparticles. J. Mater. Res. Technol..

[49] Gowtham H.G., Shilpa N., Singh S.B., Aiyaz M., Abhilash M.R., Nataraj K., Murali M. (2024). Toxicological Effects of Nanoparticles in Plants: Mechanisms Involved at Morphological, Physiological, Biochemical and Molecular Levels. Plant Physiol. Biochem..

[50] Khan F., Shariq M., Asif M., Siddiqui M.A., Malan P., Ahmad F. (2022). Green Nanotechnology: Plant-Mediated Nanoparticle Synthesis and Application. Nanomaterials.

[51] Ahmad M.A., Adeel M., Shakoor N., Ali I., Ishfaq M., Haider F.U., Deng X. (2023). Unraveling the Roles of Modified Nanomaterials in Nano-Enabled Agriculture. Plant Physiol. Biochem..

[52] Paramo L.A., Feregrino-Pérez A.A., Guevara R., Mendoza S., Esquivel K. (2020). Nanoparticles in Agroindustry: Applications, Toxicity, Challenges, and Trends. Nanomaterials.

[53] Yaqoob A.A., Umar K., Ibrahim M.N.M. (2020). Silver Nanoparticles: Various Methods of Synthesis, Size Affecting Factors and Their Potential Applications—A Review. Appl. Nanosci..

[54] El Gamal A.Y., Tohamy M.R., I Abou-Zaid M., Atia M.M., El Sayed T., Farroh K.Y. (2022). Silver Nanoparticles as a Viricidal Agent to Inhibit Plant-Infecting Viruses and Disrupt Their Acquisition and Transmission by Their Aphid Vector. Arch. Virol..

[55] De Jesús Rivero-Montejo S., Rivera-Bustamante R.F., Saavedra-Trejo D.L., Vargas-Hernandez M., Palos-Barba V., Macias-Bobadilla I., Torres-Pacheco I. (2023). Inhibition of Pepper Huasteco Yellow Veins Virus by Foliar Application of ZnO Nanoparticles in *Capsicum annuum* L.. Plant Physiol. Biochem..

[56] Aseel D.G., Rabie M., El-Far A., Abdelkhalek A. (2024). Antiviral Properties and Molecular Docking Studies of Eco-Friendly Biosynthesized Copper Oxide Nanoparticles against Alfalfa Mosaic Virus. BMC Plant Biol..

[57] Al-Zaban M.I., Alhag S.K., Dablool A.S., Ahmed A.E., Alghamdi S., Ali B., Al-Saeed F.A., Saleem M.H., Poczai P. (2022). Manufactured Nano-Objects Confer Viral Protection against Cucurbit Chlorotic Yellows Virus (CCYV) Infecting *Nicotiana benthamiana*. Microorganisms.

[58] Mascia T., Nigro F., Abdallah A., Ferrara M., De Stradis R., Faedda P., Palukaitis P., Gallitelli D. (2014). Gene Silencing and Gene Expression in Phytopathogenic Fungi Using a Plant Virus Vector. Proc. Natl. Acad. Sci. USA.

[59] Garcia-Ruiz H., Carbonell A., Hoyer J.S., Fahlgren N., Gilbert K.B., Takeda A., Giampetruzzi A., Ruiz M.T.G., McGinn M.G., Lowery N. (2015). Roles and Programming of Arabidopsis ARGONAUTE Proteins during Turnip Mosaic Virus Infection. PLoS Pathog..

[60] Parperides E., El Mounadi K., Garcia-Ruiz H. (2023). Induction and Suppression of Gene Silencing in Plants by Nonviral Microbes. Mol. Plant Pathol..

[61] Voinnet O. (2005). Induction and Suppression of RNA Silencing: Insights from Viral Infections. Nat. Rev. Genet..

[62] Kocken J.M.M. (2024). Molecular Directors: Non-Coding RNA and Extracellular Vehicle in Right Ventricle Remodeling. J. Mol. Med..

[63] Wang Z., Cao S., Xu X., He Y., Shou W., Munaiz E.D., Yu C., Shen J. (2023). Application and Expansion of Virus-Induced Gene Silencing for Functional Studies in Vegetables. Horticulturae.

[64] Tomari Y., Zamore P.D. (2005). Perspective: Machines for RNAi. Genes Dev..

[65] Zeng Y., Cullen B.R. (2002). RNA Interference in Human Cells Is Restricted to the Cytoplasm. RNA.

[66] Montgomery T.A., Howell M.D., Cuperus J.T., Li D., Hansen J.E., Alexander A.L., Chapman E.J., Fahlgren N., Allen E., Carrington J.C. (2008). Specificity of ARGONAUTE7-miR390 Interaction and Dual Functionality in TAS3 Trans-Acting siRNA Formation. Cell.

[67] Cuperus J.T., Carbonell A., Fahlgren N., Garcia-Ruiz H., Burke R.T., Takeda A., Sullivan C.M., Gilbert S.D., Montgomery T.A., Carrington J.C. (2010). Unique Functionality of 22-nt miRNAs in Triggering RDR6-Dependent siRNA Biogenesis from Target Transcripts in *Arabidopsis*. Nat. Struct. Mol. Biol..

[68] Zulfiqar S., Farooq M.A., Zhao T., Wang P., Tabusam J., Wang Y., Xuan S., Zhao J., Chen X., Shen S. (2023). Virus-Induced Gene Silencing (VIGS): A Powerful Tool for Crop Improvement and Its Advancement towards Epigenetics. Int. J. Mol. Sci..

[69] Huang C., Qian Y., Li Z., Zhou X. (2012). Virus-Induced Gene Silencing and Its Application in Plant Functional Genomics. Sci. China Life Sci..

[70] Zhao J., Yan S., Li M., Sun L., Dong M., Yin M., Shen J., Zhao Z. (2023). NPFR Regulates the Synthesis and Metabolism of Lipids and Glycogen via AMPK: Novel Targets for Efficient Corn Borer Management. Int. J. Biol. Macromol..

[71] Venu E., Ramya A., Babu P.L., Srinivas B., Kumar S., Reddy N.K., Babu Y.M., Majumdar A., Manik S. (2025). Exogenous dsRNA-Mediated RNAi: Mechanisms, Applications, Delivery Methods and Challenges in the Induction of Viral Disease Resistance in Plants. Viruses.

[72] Ruiz M.T., Voinnet O., Baulcombe D.C. (1998). Initiation and Maintenance of Virus-Induced Gene Silencing. Plant Cell.

[73] Shi J., Liu W., Fu Y., Yin N., Zhang H., Chang J., Zhang Z. (2018). “US-Detonated Nano Bombs” Facilitate Targeting Treatment of Resistant Breast Cancer. J. Control. Release.

[74] Valentine T., Shaw J., Blok V.C., Phillips M.S., Oparka K.J., Lacomme C. (2004). Efficient Virus-Induced Gene Silencing in Roots Using a Modified Tobacco Rattle Virus Vector. Plant Physiol..

[75] Yin Y., Zhao P., Sun Y., Han T., Wang M., Yan M., Wu J., Zhou H., Ye J. (2025). Development and Application of Sugarcane Yellow Leaf Virus Vectors in Sugarcane. Phytopathol. Res..

[76] Gaafar Y.Z.A., Ziebell H. (2020). Novel Targets for Engineering Physostegia Chlorotic Mottle and Tomato Brown Rugose Fruit Virus-Resistant Tomatoes: In Silico Prediction of Tomato MicroRNA Targets. PeerJ.

[77] Ameres S.L., Martinez J., Schroeder R. (2007). Molecular Basis for Target RNA Recognition and Cleavage by Human RISC. Cell.

[78] Akbar S., Tahir M., Wang M.B., Liu Q. (2017). Expression Analysis of Hairpin RNA Carrying Sugarcane Mosaic Virus (SCMV) Derived Sequences and Transgenic Resistance Development in a Model Rice Plant. Biomed. Res. Int..

[79] Qi T., Guo J., Peng H., Liu P., Kang Z., Guo J. (2019). Host-Induced Gene Silencing: A Powerful Strategy to Control Diseases of Wheat and Barley. Int. J. Mol. Sci..

[80] Harvey J.J., Lewsey M.G., Patel K., Westwood J., Heimstädt S., Carr J.P., Baulcombe D.C. (2011). An Antiviral Defense Role of AGO2 in Plants. PLoS ONE.

[81] Fahim M., Millar A.A., Wood C.C., Larkin P.J. (2012). Resistance to Wheat Streak Mosaic Virus Generated by Expression of an Artificial Polycistronic MicroRNA in Wheat. Plant Biotechnol. J..

[82] Wassenegger M., Krczal G. (2006). Nomenclature and Functions of RNA-Directed RNA Polymerases. Trends Plant Sci..

[83] Boutet S., Vazquez F., Liu J., Béclin C., Fagard M., Gratias A., Morel J.B., Crété P., Chen X., Vaucheret H. (2003). Arabidopsis HEN1: A Genetic Link between Endogenous miRNA Controlling Development and siRNA Controlling Transgene Silencing and Virus Resistance. Curr. Biol..

[84] Garcia Ruiz M.T., Knapp A.N., Garcia-Ruiz H. (2018). Profile of Genetically Modified Plants Authorized in Mexico. GM Crops Food.

[85] Kwon J., Kasai A., Maoka T., Masuta C., Sano T., Nakahara K.S. (2020). RNA Silencing-Related Genes Contribute to Tolerance of Infection with Potato Virus X and Y in a Susceptible Tomato Plant. Virol. J..

[86] Cisneros A., Martín-García T., Primc A., Orlando F., Gomez J.J.L., Kuziuta W., Espadas A.P. (2023). Transgene-Free, Virus-Based Gene Silencing in Plants by Artificial Small RNAs Derived from Minimal Precursors. Nucleic Acids Res..

[87] Vetukuri R.R., Dubey M., Kalyandurg P.B., Carlsson A.S., Whisson S.C., Ortiz R. (2021). Spray-Induced Gene Silencing: An Innovative Strategy for Plant Trait Improvement and Disease Control. Crop Breed. Appl. Biotechnol..

[88] Dubrovina A.S., Kiselev K.V. (2019). Exogenous RNAs for Gene Regulation and Plant Resistance. Int. J. Mol. Sci..

[89] Kasi Viswanath K., Hamid A., Ateka E., Pappu H.R. (2023). CRISPR/Cas, Multiomics, and RNA Interference in Virus Disease Management. Phytopathology®.

[90] Vaddoriya H.K., Vaddoriya H.K., Shelar V.B., Pampaniya A., Panwala A. (2024). “BioClay™”: One Step toward the Sustainable and Novel Plant Protection Method. Int. J. Econ. Plants.

[91] Worrall E.A., Bravo-Cazar A., Nilon A.T., Fletcher S.J., Robinson K.E., Carr J.P., Mitter N. (2019). Exogenous Application of RNAi-Inducing Double-Stranded RNA Inhibits Aphid-Mediated Transmission of a Plant Virus. Front. Plant Sci..

[92] Qiao H., Chen J., Dong M., Shen J., Yan S. (2024). Nanocarrier-Based Eco-Friendly RNA Pesticides for Sustainable Management of Plant Pathogens and Pests. Nanomaterials.

[93] Chariou P.L., Ortega-Rivera O.A., Steinmetz N.F. (2020). Nanocarriers for the Delivery of Medical, Veterinary, and Agricultural Active Ingredients. ACS Nano.

[94] Vogel E., Santos D., Mingels L., Verdonckt T.-W., Broeck J.V. (2019). RNA Interference in Insects: Protecting Beneficials and Controlling Pests. Front. Physiol..

[95] Sangwan A., Gupta D., Singh O.W., Roy A., Mukherjee S.K., Mandal B., Singh N. (2023). Size Variations of Mesoporous Silica Nanoparticle Control Uptake Efficiency and Delivery of AC2-Derived dsRNA for Protection against Tomato Leaf Curl New Delhi Virus. Plant Cell Rep..

[96] Miyamoto T., Numata K. (2023). Advancing Biomolecule Delivery in Plants: Harnessing Synthetic Nanocarriers to Overcome Multiscale Barriers for Cutting-Edge Plant Bioengineering. Bull. Chem. Soc. Jpn..

[97] Yoon J.-S., Gurusamy D., Pali S.R. (2017). Accumulation of dsRNA in Endosomes Contributes to Inefficient RNA Interference in the Fall Armyworm, *Spodoptera frugiperda*. Insect Biochem. Mol. Biol..

[98] Fu Y., Li L., Wang H., Jiang Y., Liu H., Cui X., Wang P., Lü C. (2015). Silica Nanoparticles-Mediated Stable Genetic Transformation in *Nicotiana tabacum*. Chem. Res. Chin. Univ..

[99] Karimi M., Ghasemi A., Sahandi Zangabad P., Rahighi R., Moosavi Basri S.M., Mirshekari H., Amiri M., Shafaei Pishabad Z., Aslani A., Bozorgomid M. (2016). Smart Micro/Nanoparticles in Stimulus-Responsive Drug/Gene Delivery Systems. Chem. Soc. Rev..

[100] Kolge H., Kadam K., Ghormade V. (2023). Chitosan Nanocarriers Mediated dsRNA Delivery in Gene Silencing for *Helicoverpa armigera* Biocontrol. Pestic. Biochem. Physiol..

[101] Ma Y.F., Liu T.T., Zhao Y.Q., Luo J., Feng H.Y., Zhou Y.Y., He P. (2024). RNA Interference-Screening of Potentially Lethal Gene Targets in the White-Backed Planthopper *Sogatella furcifera* via a Spray-Induced and Nanocarrier-Delivered Gene Silencing System. J. Agric. Food Chem..

[102] Zheng W., Xu X., Huang X., Peng J., Ma W., Hull J.J., Hua H., Chen L. (2024). Spray-Induced and Nanocarrier-Delivered Gene Silencing System Targeting Juvenile Hormone Receptor Components: Potential Application as Fertility Inhibitors for *Adelphocoris suturalis* Management. Pest Manag. Sci..

[103] Lv H., Li X., Li J., Yu C., Zeng Q., Ning G., He S. (2023). Overcoming Resistance in Insect Pest with a Nanoparticle-Mediated dsRNA and Insecticide Co-Delivery System. Chem. Eng. J..

[104] Wang K., Peng Y., Chen J., Peng Y., Wang X., Shen Z., Han Z. (2020). Comparison of Efficacy of RNAi Mediated by Various Nanoparticles in the Rice Striped Stem Borer (*Chilo suppressalis*). Pestic. Biochem. Physiol..

[105] Cooper A.M., Song H., Yu Z., Biondi M., Bai J., Shi X., Ren Z., Weerasekara S.M., Hua D.H., Silver K. (2021). Comparison of Strategies for Enhancing RNA Interference Efficiency in *Ostrinia nubilalis*. Pest Manag. Sci..

[106] Gurusamy D., Mogilicherla K., Shukla J.N., Palli S.R. (2020). Lipids Help Double-Stranded RNA in Endosomal Escape and Improve RNA Interference in the Fall Armyworm, *Spodoptera frugiperda*. Arch. Insect Biochem. Physiol..

[107] Yan S., Qian J., Cai C., Ma Z., Li J., Yin M., Ren B., Shen J. (2019). Spray Method Application of Transdermal dsRNA Delivery System for Efficient Gene Silencing and Pest Control on Soybean Aphid *Aphis glycines*. J. Pest Sci..

[108] Sun Y., Wang P., Abouzaid M., Zhou H., Liu H., Yang P., Lin Y., Hull J.J., Ma W. (2020). Nanomaterial-Wrapped dsCYP15C1, a Potential RNAi-Based Strategy for Pest Control against *Chilo suppressalis*. Pest Manag. Sci..

[109] Laisney J., Gurusamy D., Baddar Z.E., Palli S.R., Unrine J.M. (2020). RNAi in *Spodoptera frugiperda* Sf9 Cells via Nanomaterial-Mediated Delivery of dsRNA: A Comparison of Poly-l-Arginine Polyplexes and Poly-l-Arginine-Functionalized Au Nanoparticles. ACS Appl. Mater. Interfaces.

[110] Martin-Ortigosa S., Peterson D.J., Valenstein J.S., Lin V.S.-Y., Trewyn B.G., Lyznik L.A., Wang K. (2014). Mesoporous Silica Nanoparticle-Mediated Intracellular Cre Protein Delivery for Maize Genome Editing via loxP Site Excision. Plant Physiol..

[111] Imran M., Feng X., Sun Z., Al Omari H., Zhang G., Zhu J., Aldayel M.F., Li C. (2025). Nanotechnology-Driven Gene Silencing: Advancements in SIGS–dsRNA Technology for Sustainable Disease Management. Chem. Biol. Technol. Agric..

[112] Park M.G., Choi J.Y., Park D.H., Wang M., Kim H.J., Je Y.H. (2021). Simultaneous Control of Sacbrood Virus (SBV) and *Galleria mellonella* Using a Bt Strain Transformed to Produce dsRNA Targeting the SBV vp1 Gene. Entomol. Gen..

[113] Guan R., Chu D., Han X., Miao X., Li H. (2021). Advances in the Development of Microbial Double-Stranded RNA Production Systems for Application of RNA Interference in Agricultural Pest Control. Front. Bioeng. Biotechnol..

[114] Low Y.C., Lawton M.A., Di R. (2022). Ethylene Insensitive 2 (EIN2) as a Potential Target Gene to Enhance Fusarium Head Blight Disease Resistance. Plant Sci..

[115] Lu Y., Deng X., Zhu Q., Wu D., Zhong J., Wen L., Yu X. (2023). The dsRNA Delivery, Targeting and Application in Pest Control. Agronomy.

[116] Yang P., Yi S.Y., Nian J.N., Yuan Q.S., He W.J., Zhang J.B., Liao Y.C. (2021). Application of Double-Strand RNAs Targeting Chitin Synthase, Glucan Synthase, and Protein Kinase Reduces *Fusarium graminearum* Spreading in Wheat. Front. Microbiol..

[117] Scarpin D., Nerva L., Chitarra W., Moffa L., D’Este F., Vuerich M., Filippi A., Braidot E., Petrussa E. (2023). Characterisation and Functionalisation of Chitosan Nanoparticles as Carriers for Double-Stranded RNA (dsRNA) Molecules towards Sustainable Crop Protection. Biosci. Rep..

[118] Wytinck N., Manchur C.L., Li V.H., Whyard S., Belmonte M.F. (2020). dsRNA Uptake in Plant Pests and Pathogens: Insights into RNAi-Based Insect and Fungal Control Technology. Plants.

[119] Wang Z., Li Y., Zhang B., Gao X., Shi M., Zhang S., Zhong S., Zheng Y., Liu X. (2023). Functionalized Carbon Dot-Delivered RNA Nano Fungicides as Superior Tools to Control *Phytophthora* Pathogens through Plant RdRP1 Mediated Spray-Induced Gene Silencing. Adv. Funct. Mater..

[120] Wang Y., Yan Q., Lan C., Tang T., Wang K., Shen J., Niu D. (2023). Nanoparticle Carriers Enhance RNA Stability and Uptake Efficiency and Prolong the Protection against *Rhizoctonia solani*. Phytopathol. Res..

[121] Yan C., Wang Y., Guo Q., Huan H., Wang S., Fan X., Xie X. (2025). Silencing Arbuscular Mycorrhizal Fungal Gene Using Chitosan Nanoparticle-Mediated dsRNA Delivery System. Bio-Protocol.

[122] Spada M., Pugliesi C., Fambrini M., Palpacelli D., Caneo A., Pecchia S. (2025). Spray-Induced Gene Silencing (SIGS): Nanocarrier-Mediated dsRNA Delivery Improves RNAi Efficiency in the Management of Lettuce Gray Mold Caused by *Botrytis cinerea*. Agronomy.

[123] Ding X., Li X., Li Y., Guo H., Cao K., An M., Zhang C., Wu Y., Liu H., Zhou R. (2025). A Novel Self-Assembled Nanocarrier-Mediated dsRNA Fungicide for Broad-Spectrum Management of *Rhizoctonia solani*. Mater. Today Bio.

[124] Tan J.A., Jones M.G., Fosu-Nyarko J. (2013). Gene Silencing in Root Lesion Nematodes (*Pratylenchus* spp.) Significantly Reduces Reproduction in a Plant Host. Exp. Parasitol..

[125] Zhang W., Wang R., Li Y., Li D., Wang X., Wen X., Feng Y., Liu Z., Ma S., Zhang X. (2025). Engineered Pine Endophytic Fungus Expressing Double-Stranded RNA Targeting Lethal Genes to Control the Plant-Parasitic Nematode *Bursaphelenchus xylophilus*. Phytopathology.

[126] Qi J., Li Y., Yao X., Li G., Xu W., Chen L., Xie Z., Gu J., Wu H., Li Z. (2024). Rational Design of ROS Scavenging and Fluorescent Gold Nanoparticles to Deliver siRNA to Improve Plant Resistance to *Pseudomonas syringae*. J. Nanobiotechnol..

[127] Xu X., Jiao Y., Shen L., Li Y., Mei Y., Yang W., Li C., Cao Y., Chen F., Li B. (2023). Nanoparticle-dsRNA Treatment of Pollen and Root Systems of Diseased Plants Effectively Reduces the Rate of Tobacco Mosaic Virus in Contemporary Seeds. ACS Appl. Mater. Interfaces.

[128] Xu X., Yu T., Zhang D., Song H., Huang K., Wang Y., Shen L., Li Y., Wang F., Zhang S. (2023). Evaluation of the Anti-Viral Efficacy of Three Different dsRNA Nanoparticles against Potato Virus Y Using Various Delivery Methods. Ecotoxicol. Environ. Saf..

[129] Wang J., Hao K., Yu F., Shen L., Wang F., Yang J., Su C. (2022). Field Application of Nanoliposomes Delivered Quercetin by Inhibiting Specific hsp70 Gene Expression against Plant Virus Disease. J. Nanobiotechnol..

[130] Numata K., Ohtani M., Yoshizumi T., Demura T., Kodama Y. (2014). Local Gene Silencing in Plants via Synthetic dsRNA and Carrier Peptide. Plant Biotechnol. J..

[131] Kolge H., Kadam K., Galande S., Lanjekar V., Ghormade V. (2021). New Frontiers in Pest Control: Chitosan Nanoparticles-Shielded dsRNA as an Effective Topical RNAi Spray for Gram Podborer Biocontrol. ACS Appl. Bio Mater..

[132] Zhou H., Wan F., Jian Y., Guo F., Zhang M., Shi S., Yang L., Li S., Liu Y., Ding W. (2023). Chitosan/dsRNA Polyplex Nanoparticles Advance Environmental RNA Interference Efficiency through Activating Clathrin-Dependent Endocytosis. Int. J. Biol. Macromol..

[133] Keppanan R., Karuppannasamy A., Nagaraja B.C., Thiruvengadam V., Kesavan S., Dhawane Y.A., Ramasamy A. (2024). Effectiveness of Chitosan Nanohydrogel Mediated Encapsulation of EcR dsRNA against the Whitefly, *Bemisia tabaci* Asia-I (Gennedius) (Hemiptera: Aleyrodidae). Pestic. Biochem. Physiol..

[134] Zheng Y., Hu Y., Yan S., Zhou H., Song D., Yin M. (2019). A Polymer/Detergent Formulation Improves dsRNA Penetration through the Body Wall and RNAi-Induced Mortality in the Soybean Aphid *Aphis glycines*. Pest Manag. Sci..

[135] Christiaens O., Swevers L., Smagghe G. (2014). dsRNA Degradation in the Pea Aphid (*Acyrthosiphon pisum*) Associated with Lack of Response in RNAi Feeding and Injection Assay. Peptides.

[136] Zheng Y., Moorlach B., Jakobs-Schönwandt D., Patel A., Pastacaldi C., Jacob S., Sede A.R., Heinlein M., Poranen M.M., Kogel K.-H. (2025). Exogenous dsRNA Triggers Sequence-Specific RNAi and Fungal Stress Responses to Control *Magnaporthe oryzae* in *Brachypodium distachyon*. Commun. Biol..

[137] Pal G., Ingole K.D., Yavvari P.S., Verma P., Kumari A., Chauhan C., Chaudhary D., Srivastava A., Bajaj A., Vemanna R.S. (2024). Exogenous Application of Nanocarrier-Mediated Double-Stranded RNA Manipulates Physiological Traits and Defence Response against Bacterial Diseases. Mol. Plant Pathol..

[138] Opdensteinen P., Caparco A.A., Steinmetz N.F. (2025). Protein-Based Spherical Nanoparticles for dsRNA Delivery to Nematodes—A Platform Technology for RNA Silencing. Mater. Today.

[139] Avital A., Muzika N.S., Persky Z., Bar G., Michaeli Y., Fridman Y., Karny A., Shklover J., Shainsky J., Savaldi-Goldstein S. (2021). Foliar Delivery of siRNA Particles for Treating Viral Infections in Agricultural Grapevines. Adv. Funct. Mater..

[140] Mosa M.A., Youssef K. (2021). Topical Delivery of Host Induced RNAi Silencing by Layered Double Hydroxide Nanosheets: An Efficient Tool to Decipher Pathogenicity Gene Function of *Fusarium* Crown and Root Rot in Tomato. Physiol. Mol. Plant Pathol..

[141] Ma Z., Zheng Y., Chao Z., Chen H., Zhang Y., Yin M., Shen J., Yan S. (2022). Visualization of the Process of a Nanocarrier-Mediated Gene Delivery: Stabilization, Endocytosis and Endosomal Escape of Genes for Intracellular Spreading. J. Nanobiotechnol..

[142] Shidore T., Zuverza-Mena N., White J.C., da Silva W. (2021). Nanoenabled Delivery of RNA Molecules for Prolonged Antiviral Protection in Crop Plants: A Review. ACS Appl. Nano Mater..

[143] Whitfield R., Anastasaki A., Truong N.P., Cook A.B., Omedes-Pujol M., Loczenski Rose V., Haddleton D.M. (2018). Efficient Binding, Protection, and Self-Release of dsRNA in Soil by Linear and Star Cationic Polymers. ACS Macro Lett..

[144] Borowski L.S., Dziembowski A., Hejnowicz M.S., Stepien P.P., Szczesny R.J. (2013). Human Mitochondrial RNA Decay Mediated by PNPase–hSuv3 Complex Takes Place in Distinct Foci. Nucleic Acids Res..

[145] Merino S., Martín C., Kostarelos K., Prato M., Vázquez E. (2015). Nanocomposite Hydrogels: 3D Polymer-Nanoparticle Synergies for On-Demand Drug Delivery. ACS Nano.

[146] Deng L., Albertazzi L., Palmans A.R.A. (2022). Elucidating the Stability of Single-Chain Polymeric Nanoparticles in Biological Media and Living Cells. Biomacromolecules.

[147] Zhang X., Zhang J., Zhu K. (2010). Chitosan/Double-Stranded RNA Nanoparticle-Mediated RNA Interference to Silence Chitin Synthase Genes through Larval Feeding in the African Malaria Mosquito (*Anopheles gambiae*). Insect Mol. Biol..

[148] Benny O., Fainaru O., Adini A., Cassiola F., Bazinet L., Adini I., Pravda E., Nahmias Y., Koirala S., Corfas G. (2008). An Orally Delivered Small-Molecule Formulation with Antiangiogenic and Anticancer Activity. Nat. Biotechnol..

[149] Chen L., Okuda T., Lu X.Y., Chan H.K. (2016). Amorphous Powders for Inhalation Drug Delivery. Adv. Drug Deliv. Rev..

[150] Zhou J., Shum K.T., Burnett J.C., Rossi J.J. (2013). Nanoparticle-Based Delivery of RNAi Therapeutics: Progress and Challenges. Pharmaceuticals.

[151] Benjaminsen R.V., Mattebjerg M.A., Henriksen J.R., Moghimi S.M., Andresen T.L. (2013). The Possible “Proton Sponge” Effect of Polyethylenimine (PEI) Does Not Include Change in Lysosomal pH. Mol. Ther..

[152] Sandonas L.M., Sevinçli H., Gutierrez R., Cuniberti G. (2018). First-Principle-Based Phonon Transport Properties of Nanoscale Graphene Grain Boundaries. Adv. Sci..

[153] Yu C., Li L., Hu P., Yang Y., Wei W., Deng X., Wang L., Tay F.R., Ma J. (2021). Recent Advances in Stimulus-Responsive Nanocarriers for Gene Therapy. Adv. Sci..

[154] Ke X., Yang C., Cheng W., Yang Y.Y. (2018). Delivery of NF-κB shRNA Using Carbamate-Mannose Modified PEI for Eliminating Cancer Stem Cells. Nanomedicine.

[155] Wang Z., Liu G., Zheng H., Chen X. (2014). Rigid Nanoparticle-Based Delivery of Anti-Cancer siRNA: Challenges and Opportunities. Biotechnol. Adv..

[156] Tzeng S.Y., Green J.J. (2013). Subtle Changes to Polymer Structure and Degradation Mechanism Enable Highly Effective Nanoparticles for siRNA and DNA Delivery to Human Brain Cancer. Adv. Healthc. Mater..

[157] Naimi-Shamel N., Pourali P., Dolatabadi S. (2019). Green Synthesis of Gold Nanoparticles Using *Fusarium oxysporum* and Antibacterial Activity of Its Tetracycline Conjugant. J. Mycol. Med..

[158] Soliman M.K.Y., Abu-Elghait M., Salem S.S., Azab M.S. (2024). Multifunctional Properties of Silver and Gold Nanoparticles Synthesis by *Fusarium pseudonygamai*. Biomass Conv. Bioref..

[159] Neira-Vielma A.A., Meléndez-Ortiz H.I., García-López J.I., Sanchez-Valdes S., Cruz-Hernández M.A., Rodríguez-González J.G., Ramírez-Barrón S.N. (2022). Green Synthesis of Silver Nanoparticles Using Pecan Nut (*Carya illinoinensis*) Shell Extracts and Evaluation of Their Antimicrobial Activity. Antibiotics.

[160] Yadav A., Mathan J., Dubey A.K., Singh A. (2024). The Emerging Role of Non-Coding RNAs (ncRNAs) in Plant Growth, Development, and Stress Response Signaling. Non-Coding RNA.

[161] Taliansky M., Samarskaya V., Zavriev S.K., Fesenko I., Kalinina N.O., Love A.J. (2021). RNA-Based Technologies for Engineering Plant Virus Resistance. Plants.

[162] Zhang H., Guo H., Hu W., Ji W. (2020). The Emerging Role of Long Non-Coding RNAs in Plant Defense Against Fungal Stress. Int. J. Mol. Sci..

[163] Khoo Y.W., Wang Q., Liu S., Zhan B., Xu T., Lv W., Liu G., Li S., Zhang Z. (2024). Resistance of the CRISPR-Cas13a Gene-Editing System to Potato Spindle Tuber Viroid Infection in Tomato and *Nicotiana benthamiana*. Viruses.

[164] Zhan X., Zhang F., Li N., Xu K., Wang X., Gao S., Yin Y., Yuan W., Chen W., Ren Z. (2024). CRISPR/Cas: An Emerging Toolbox for Engineering Virus Resistance in Plants. Plants.

[165] Ding J., Cui C., Wang G., Wei G., Bai L., Li Y., Sun P., Dong L., Liu Z., Yun J. (2023). Engineered Gut Symbiotic Bacterium-Mediated RNAi for Effective Control of *Anopheles* Mosquito Larvae. Microbiol. Spectr..

